# Supplementary material for: The SPOC proteins DIDO3 and PHF3 co-regulate gene expression and neuronal differentiation
Source: Nat Commun. 2023 Nov 30;14:7912. doi: 10.1038/s41467-023-43724-y (PMC10689479; doi:10.1038/s41467-023-43724-y)
Supplement: Supplementary file 1 — Supplementary Information [file 41467_2023_43724_MOESM1_ESM.pdf]

SUPPLEMENTARY INFORMATION FOR

The SPOC proteins DIDO3 and PHF3 co-regulate gene expression and neuronal differentiation

Johannes Benedum[1,2,3,4], Vedran Franke[5], Lisa-Marie Appel[1,2,3], Lena Walch[1], Melania Bruno[1], Rebecca Schneeweiss[1], Juliane Gruber[1], Helena Oberndorfer[1], Emma Frank[1], Xué Strobl[1,4], Anton Polyansky[6], Bojan Zagrovic[6], Altuna Akalin[5], Dea Slade[1,2,3,*]

[1]Department of Medical Biochemistry, Medical University of Vienna, Max Perutz Labs, Vienna Biocenter, 1030 Vienna, Austria

[2]Department of Radiation Oncology, Medical University of Vienna, Währinger Gürtel 18-20, 1090 Vienna, Austria

[3]Comprehensive Cancer Center, Medical University of Vienna, Spitalgasse 23, 1090 Vienna, Austria

[4]Vienna Biocenter PhD Program, a Doctoral School of the University of Vienna and Medical University of Vienna, 1030, Vienna, Austria.

[5]The Berlin Institute for Medical Systems Biology, Max Delbrück Center, Robert-Rössle-Straße 10, 13125 Berlin, Germany

[6]Department of Structural and Computational Biology, Max Perutz Labs, University of Vienna, Vienna Biocenter, Campus Vienna Biocenter 5, 1030 Vienna, Austria

[*]Correspondence: dea.slade@maxperutzlabs.ac.at

Supplementary Tables

Supplementary Table 1. Antibodies

Antibody	Source	Identifier & dilution
Rabbit anti-CK2α	Cell Signaling	2656; 1:1000 for WB
Rabbit anti-DEK	Cell Signaling	29812; cl. E4S5J; 1:1000 for WB
Rabbit anti-DIDO1	Atlas Antibody	HPA0449904; 1:200 for IF, 1:500 for WB
Rabbit anti-Pan DIDO	Millipore	ABN1367; 1:500for WB
Mouse anti-Human DIDO1	R&D Systems	MAB6947; cl. 734823; 1:250 for WB
Mouse anti-FLAG	Sigma	F1804; cl. M2; 1:700 for IF
Mouse anti-FLAG M2-peroxidase	Sigma	A8592; 1:10000 for WB
Rabbit anti-GFP	abcam	ab290; 1:1000 for IF
Mouse anti-GFP	Roche	11814460001; 1:200 for IF
Rabbit anti-H3K9ac	Sigma	07-352; 1:1000 for IF
Rabbit anti-H3K9me3	abcam	Ab8898; 1:500 for IF
Rabbit anti-fibrillarin (C13C3)	Cell Signaling	2639; 1:200 for IF
Mouse anti-HA.11	Covance	901513; cl. 16B12; 1:1000 for WB
Rabbit anti-HCFC1	Cell Signaling	69690; 1:1000 for WB
Rabbit anti-histone macro H2A1	abcam	ab37264; 1:1000 for WB
Rabbit anti-histone macro H2A1.2	Cell Signaling	4827; 1:1000 for WB
Rabbit anti-H2AZ	Cell Signaling	2718; 1:1000 for WB
Rabbit anti-gH2AX	Bethyl	A300-081A; 1:1000 for WB
Rabbit anti-PAF1	Abcam	ab20662; 1:1000 for WB
Mouse anti-PARP C2-10	Trevigen	4338-MC-50; cl. C2-10; 1:1000 for WB
Mouse anti-PARP2	Enzo	ALX-804-639; cl. 4G8; 1:25 for WB
Rabbit anti-PHF3	Atlas antibodies	HPA025763; 1:500 for WB
Rat anti-pS2 Pol II	Monoclonal antibody facility (Helmholtz Center, Munich)	cl. 3E10; 1:25 for IF; 1:1000 for WB
Rat anti-pS5 Pol II	Monoclonal antibody facility (Helmholtz Center, Munich)	cl. 3E8; 1:1000 for WB
Rat anti-RNA polymerase II pS7 4E12	Millipore	04-1570; cl. 4E12; 1:1000 for WB
Mouse anti-pS5 Pol II clone 4H8	Cell Signaling	2629; cl. 4H8 1:1000 for WB
Mouse anti-RNA polymerase II	Santa Cruz	sc-55492; cl. F-12; 1:1000 for WB
Mouse anti-SPT5	BD	611107; cl. 17; 1:1000 for WB
Rabbit anti-SPT6	Novus Biologicals	NB100-2582; 1:1000 for WB
Mouse anti-α-tubulin	Sigma	T6074; cl. B-5-1-2; 1:5000 for WB
Rabbit anti-β-Tubulin III TuJ1	Sigma	T2200; 1:500 for IF
Mouse anti-GFAP	Sigma	G3893, 1:500 for IF
Rabbit anti-YB1	Cell Signaling	D299; 1:1000 for WB
Rat anti-ZNF768	AG Eick	cl. 5c8; 1:10 for WB
Rabbit TBP (D5C9H) XP	Cell Signalling	44059;1:1000 for WB
Goat anti-rabbit AF488	ThermoFisher	A11008; 1:500 for IF
Goat anti-mouse AF488	ThermoFisher	A11001; 1:500 for IF
Goat anti-rabbit AF568	ThermoFisher	A11011; 1:500 for IF
Goat anti-mouse AF568	ThermoFisher	A11004; 1:500 for IF
Goat anti-rat AF594	ThermoFisher	A11007; 1:500 for IF
Goat anti-rat AF647	Abcam	ab150167; 1:500 for IF
Goat anti-rabbit AF647	ThermoFisher	A21244; 1:200 for IF

Supplementary Table 2. Plasmids

Plasmid	Source
CMV10 N3XFLAG DIDO3	Appel et al. 2023
CMV10 N3XFLAG DIDO3 ΔN	This paper
CMV10 N3XFLAG DIDO2	This paper
CMV10 N3XFLAG DIDO2 ΔN	This paper
CMV10 N3XFLAG DIDO1	This paper
CMV10 N3XFLAG DIDO1 ΔN	This paper
CMV10 N3XFLAG DIDO3 ΔSPOC	Appel et al. 2023
CMV10 N3XFLAG PHF3	Appel et al. 2023
CMV10 N3XFLAG PHF3 ΔSPOC	Appel et al. 2023
CMV10 N3XFLAG PHF3 NLS-SPOC	Appel et al. 2023
CMV10 2xHA DIDO3	This paper
CMV10 2xHA DIDO3 1-1689	This paper
CMV10 2xHA DIDO3 1-2084	This paper
CMV10 2xHA DIDO3 1690-1956	This paper
CMV10 2xHA DIDO3 2052-2240	This paper
CMV10 2xHA DIDO3 1690-2240	This paper
CMV10 2xHA DIDO3 1690-1798	This paper
CMV10 2xHA DIDO3 1768-1956	This paper
CMV10 2xHA DIDO3 2085-2240	This paper
CMV10 N3XFLAG PHF3 1-1594	This paper
CMV10 N3XFLAG PHF3 1595-2039	This paper
pET Duet 6xHis mCherry-PHF3 1595-2039	This paper
pET Duet 6xHis mCherry-PHF3 1595-1913	This paper
pET Duet 6xHis mCherry-PHF3 1595-1795	This paper
pET Duet 6xHis mCherry-PHF3 1796-2039	This paper
pET Duet 6xHis mCherry-PHF3 1796-1887	This paper
pET Duet 6xHis mCherry-PHF3 1888-2039	This paper
pET Duet 6xHis mCherry-DIDO 1690-2240	This paper
mEGFP-DIDO 1690-1956	This paper
mEGFP-DIDO 1690-1798	This paper
mEGFP-DIDO 1768-1956	This paper
mEGFP-DIDO 2052-2240	This paper
mEGFP-DIDO 2085-2240	This paper
Cas9-EGFP (PX458) DIDO N[1-88]-Isoform KO gRNA	This paper
Cas9-EGFP (PX458) DIDO Long Isoform KO gRNA	This paper
Cas9-EGFP (PX458) DIDO Full KO gRNA	This paper
Cas9-EGFP (PX458) DIDO ΔSPOC gRNA1	This paper
Cas9-EGFP (PX458) DIDO ΔSPOC gRNA2	This paper
Cas9-EGFP (PX458) DIDO ΔIDR gRNA1	This paper
Cas9-EGFP (PX458) DIDO ΔIDR gRNA2	This paper
Cas9 (PX330) DIDO 3' gRNA	This paper
Cas9 (pX461) PHF3 3' gRNA 1	Appel et al. 2021
Cas9 (pX461) PHF3 3' gRNA 2	Appel et al. 2021
Cas9 (pX458) PHF3 ΔIDR gRNA	This paper
pUC19 AID-EGFP-P2A_puromycin repair template for DIDO-AID-GFP tagging	This paper
p1559 LoxP-SFFV-Hygromycin-LoxP repair template for DIDO SPOC deletion	This paper
p1561 LoxP-SFFV-Puromycin-LoxP repair template for DIDO SPOC deletion	This paper
p1560 LoxP-SFFV-Blasticitin-LoxP repair template for DIDO SPOC deletion	This paper
pUC19 PHF3 ΔIDR-mAID-3xFLAG-mScarlet-P2A-Puromycin repair template for PHF3 IDR deletion	This paper

Supplementary Table 3. Cell lines

Cell line	Source	Identifier
HEK293T	ATCC CRL-3216	RRID:CVCL_0063
MEFs	ATCC CRL-2991	RRID: CVCL_L690
S2	ATCC CRL-1963	RRID: CVCL_Z232
HEK293T PHF3 KO	Appel et al. 2021[1]	N/A
HEK293T PHF3 ΔSPOC	Appel et al. 2021[1]	N/A
HEK293T PHF3 WT	Appel et al. 2021[1]	N/A
HEK293T PHF3-GFP	Appel et al. 2021[1]	N/A
HEK293T DIDO full KO	Appel et al. 2023[2]	DKE4-B8
HEK293T DIDO ΔSPOC	Appel et al. 2023[2]	HDDS-G7
HEK293T DIDO N[1-88]-Isoform KO	This paper	DK1-B7
HEK293T DIDO Long Isoform KO	This paper	DK2-G1
PHF3 ΔSPOC DIDO Full KO	This paper	PDSDKE4-B11
PHF3 KO DIDO Full KO	This paper	PKDKE4-A3
PHF3 ΔSPOC DIDO ΔSPOC	This paper	Δ^2-C3
PHF3 KO DIDO ΔSPOC	This paper	PKDDS-E2
DIDO ΔIDR	This paper	HDDM-B7
DIDO 3’ AID-GFP	This paper	HDAG-B8
PHF3 ΔSPOC DIDO 3’ AID-GFP	This paper	PDSDAG-C5
PHF3 KO DIDO 3’ AID-GFP	This paper	PKDAG-B7
DIDO ΔSPOC 3’ AID-GFP	This paper	HDDSAG-B10
DIDO ΔIDR 3’ AID-GFP	This paper	HDDMAG-A6
PHF3 ΔIDR-3xFLAG-mScarlet	This paper	1A11
PHF3 WT / SPOC OE	This paper	WT/NLSSPOC.OE – cl.2
PHF3 WT / PHF3 OE	This paper	WT/PHF3.OE – cl.4
PHF3 WT / PHF3 ΔSPOC OE	This paper	WT/ΔSPOC.OE – cl.2
PHF3 KO / SPOC OE	This paper	KO/NLSSPOC.OE – cl.7
PHF3 KO / PHF3 OE	This paper	KO/PHF3.OE – cl.5
PHF3 KO / PHF3 ΔSPOC OE	This paper	KO/ΔSPOC.OE – cl.7
PHF3-GFP DIDO full KO	This paper	B10
DIDO 3’ AID-GFP PHF3-3xFLAG-mScarlet	This paper	C5
Phf3 WT mESCs	Appel et al. 2021[1]	N/A
Phf3 KO mESCs	Appel et al. 2021[1]	N/A
Dido heterozygous KO mESCs	Lackner et al. 2021[3]	N/A

Supplementary Table 4. Image acquisition parameters for confocal imaging

	**Confocal**	Channel	Laser power [%]	(1) [V]	Emission range [nm]	(2) [µm] /(3) [nm]	(4) [µs]	Frame size [px]
Fig 3g<br>Fig S6d	DIDO WT vs.<br>dSPOC vs.dIDR	AF488<br>DAPI	0.2<br>0.9	650<br>700	499-552<br>410-508	50/71<br>44/71	1.87	1122x1122
Fig 3g	PHF3WT<br>vs. dIDR	AF568<br>DAPI	2.8<br>0.4	650	570-694<br>411-543	38/71<br>36/71	2.79	1503x1503
Fig 4g	H3K9me3<br>& DIDO WT vs<br>dIDR	AF647<br>AF488<br>DAPI	0.8<br>2.0<br>0.4	650<br>665<br>650	641-694<br>490-579<br>411-605	68/71<br>54/71<br>51/71	4.67	898x898
Fig 4h	H3K9me3<br>& PHF3 WT vs<br>dIDR	AF647<br>AF568<br>DAPI	2.0<br>3.5<br>0.6	650<br>700<br>650	641-694<br>570-641<br>411-605	68/71<br>61/71<br>51/71	4.67	898x898
Fig S2a	DIDO dN<br>isoforms	AF568<br>DAPI	0.2<br>2.6	650<br>700	570-694<br>410-499	60/71<br>43/71	0.47	1122x1122
Fig S15	H3K9ac<br>& DIDO WT vs<br>dIDR	AF647<br>AF488<br>DAPI	0.4<br>2.0<br>0.4	600<br>665<br>650	641-694<br>490-579<br>411-605	68/71<br>54/71<br>51/71	4.67	898x898
Fig S15	Fibrillarin<br>& DIDO WT vs<br>dIDR	AF647<br>AF488<br>DAPI	2.0<br>2.0<br>0.4	660<br>665<br>650	641-694<br>490-579<br>411-605	68/71<br>54/71<br>51/71	4.67	898x898
Fig S15	H3K9ac<br>& PHF3 WT vs<br>dIDR	AF647<br>AF568<br>DAPI	1.0<br>3.5<br>0.6	630<br>700<br>650	641-694<br>570-641<br>411-605	68/71<br>61/71<br>51/71	4.67	898x898
Fig S15	Fibrillarin<br>& PHF3 WT vs<br>dIDR	AF647<br>AF568<br>DAPI	1.5<br>3.5<br>0.6	675<br>700<br>650	641-694<br>570-641<br>411-605	68/71<br>61/71<br>51/71	4.67	898x898
Fig<br>S16d	DIDO KO<br>isoforms	AF568<br>DAPI	1.5<br>1.0	700	570-641<br>410-535	58/71<br>43/71	4.4	954x954

(1) Detector voltage; (2) Pinhole size; (3) Pixel size; (4) Pixel dwell time

Supplementary Table 5. Image acquisition parameters for Airyscan imaging

	**Airyscan**	Channel	Laser power [%]	(1) [V]	Emission range [nm]	Secondary Beam Splitters	(2) [µm] /(3) [nm]	(4) [µs]	Frame size [px]
Fig 4a	DIDO WT dSPOC dIDR vs. Pol II pS2	AF488<br>AF594	0.5<br>5.0	700	380-608<br>574-720	SBS SP 615<br>SBS LP 570	313/43	1.69	2488x2488
Fig 4c	PHF3 WT dIDR vs. Pol II pS2	AF568<br>AF647	4.0<br>3.0	700<br>750	422-477 +<br>573-627<br>630-720	BP420-480 +<br>BP570-630	357/49	1.94	2168x2168
Fig S7a	DIDO-GFP vs. Pol II pS2<br>PHF3-GFP vs. Pol II pS2	AF488<br>AF594<br>AF488<br>AF594	0.5<br>6.0<br>1.0<br>5.0	700	380-608<br>574-720	SBS SP 615<br>SBS LP 570	313/43	1.68	2492x2492
Fig S7c&e	DIDO WT dSPOC dIDR NVP-2 & Pla-B	AF488<br>AF594	0.5<br>5.0	700	380-608<br>574-720	SBS SP 615<br>SBS LP 570	313/43	1.68	2492x2492
Fig S8a	DIDO vs. Pol II (PHF3 WT KO dSPOC)	AF488<br>AF594	0.5<br>6.0	700	380-608<br>574-720	SBS SP 615<br>SBS LP 570	313/43	1.68	2492x2492
Fig S8c	PHF3 vs. Pol II (DIDO WT KO)	AF488<br>AF594	1.0<br>5.0	700	380-608<br>574-720	SBS SP 615<br>SBS LP 570	313/43	1.69	2484x2484
Fig S8e	DIDO KO in PHF3-GFP	EGFP<br>AF568	9.0<br>2.0	700	420-480 +<br>495-550<br>574-720	BP 420-480 +<br>BP 495-550<br>SBS LP 570	313/43	2.83	1484x1484
Fig S11a	DIDO-GFP<br>PHF3-Scarlet	EGFP<br>mScarlet<br>DAPI	8.0<br>15.0<br>1.0	700	420-480 +<br>495-550<br>574-720<br>350-499	BP 420-480 +<br>BP 495-550<br>SBS LP 570<br>SBS SP 505	313/35	1.40	3004x3008

(1) Detector voltage; (2) Pinhole size; (3) Pixel size; (4) Pixel dwell time

Supplementary Figures

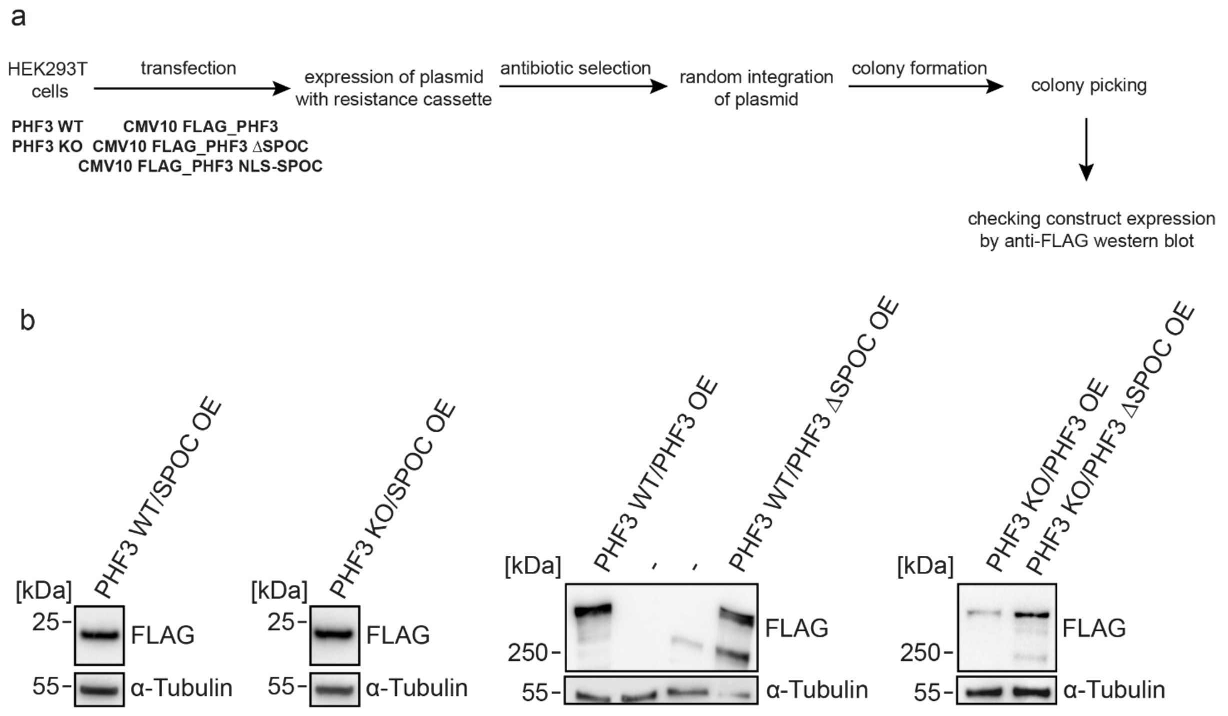

Supplementary Fig. 1: Generation of PHF3, PHF3 ΔSPOC and NLS-SPOC overexpression cell lines. a HEK293T cells were transfected with constructs bearing FLAG-PHF3, FLAG-PHF3 ΔSPOC and FLAG-NLS-SPOC (NLS = Nuclear localization signal). Hygromycin selection was used to generate stable cell lines with random integration of the constructs. **b** Integration of FLAG constructs was verified by anti-FLAG western blotting. The experiments were performed once. Source data are provided as a Source Data file.

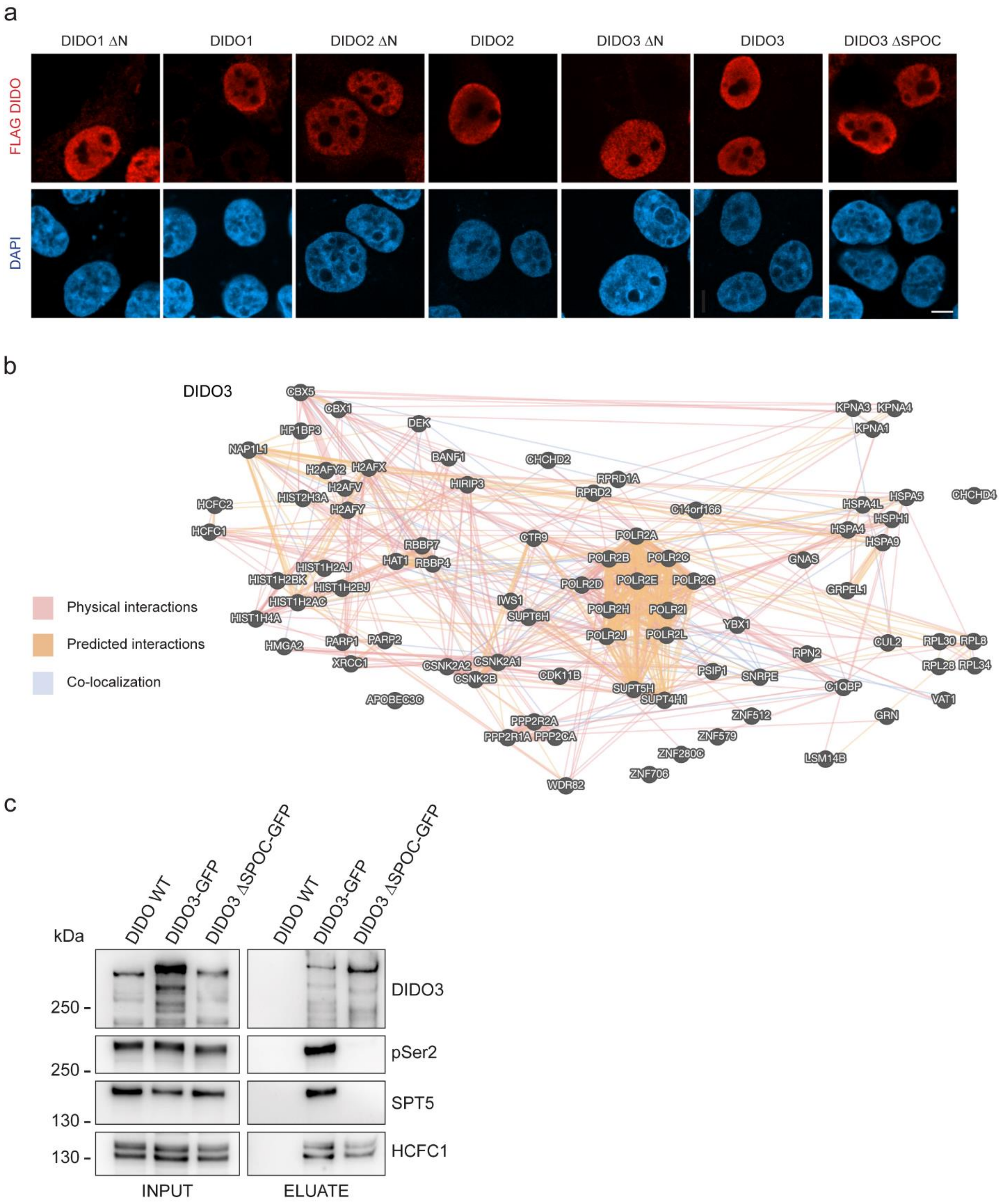

Supplementary Fig. 2: Analysis of the DIDO3 interactome by mass spectrometry. a Immunofluorescence anti-FLAG staining of FLAG-DIDO constructs transiently transfected into HEK293T cells. Scale bar=10 µm. Experiments were performed in two independent replicates, representative images are shown. **b** GeneMANIA interaction map of the DIDO3 interactome (p-value<0.05; fold change to empty vector>1.5). Mass spectrometry data are provided in Supplementary Data 1. **c** Endogenous DIDO3-GFP and DIDO3 ΔSPOC-GFP were immunoprecipitated using anti-GFP. DIDO3 interacts with Pol II and SPT5 in a SPOC-dependent manner. The experiment was performed once. Source data are provided as a Source Data file.

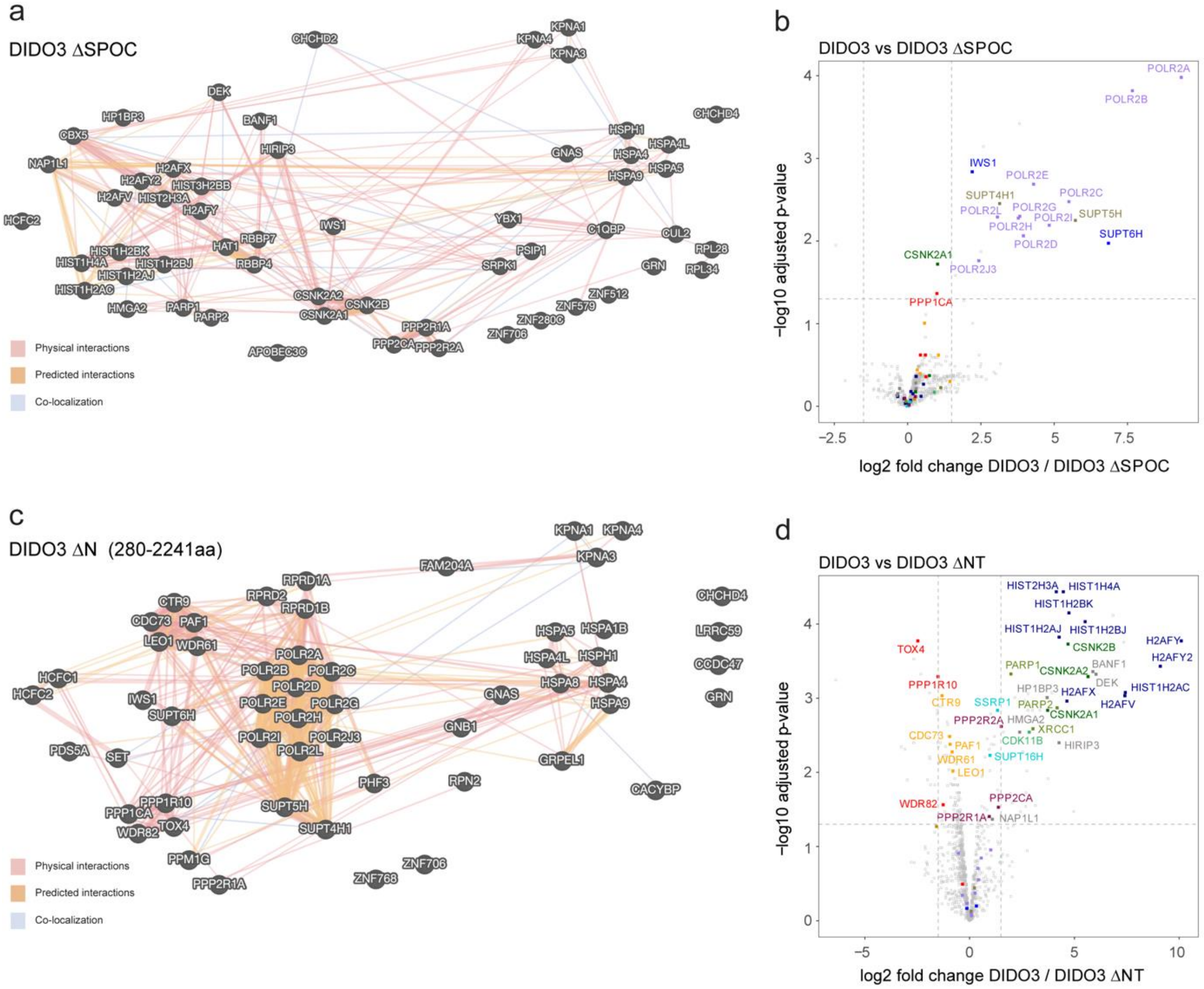

Supplementary Fig. 3: Analysis of DIDO3 ΔSPOC and ΔN interactomes by mass spectrometry. a,c GeneMANIA interaction maps for **a** DIDO ΔSPOC and **c** DIDO3 ΔN. **b,d** Volcano plots for **b** DIDO3 vs DIDO3 ΔSPOC and **d** DIDO3 vs DIDO3 ΔN. The Pol II complex is indicated in purple, SPT6/IWS1 in blue, DSIF (SPT4/SPT5) in olive, CK2 in dark green, PP1 in red, PP2A in maroon, PAF1C in orange, FACT (SPT6/SSRP1) in turquoise, histones in dark blue, chromatin-associated factors in grey, DNA damage response factors in light green. The experiments were carried out in three individual replicates. Statistical calculations were performed using the LIMMA package in R[4]. Adjusted p-values were calculated using the Benjamini-Hochberg correction for multiple testing. Mass spectrometry data are provided in Supplementary Data 1.

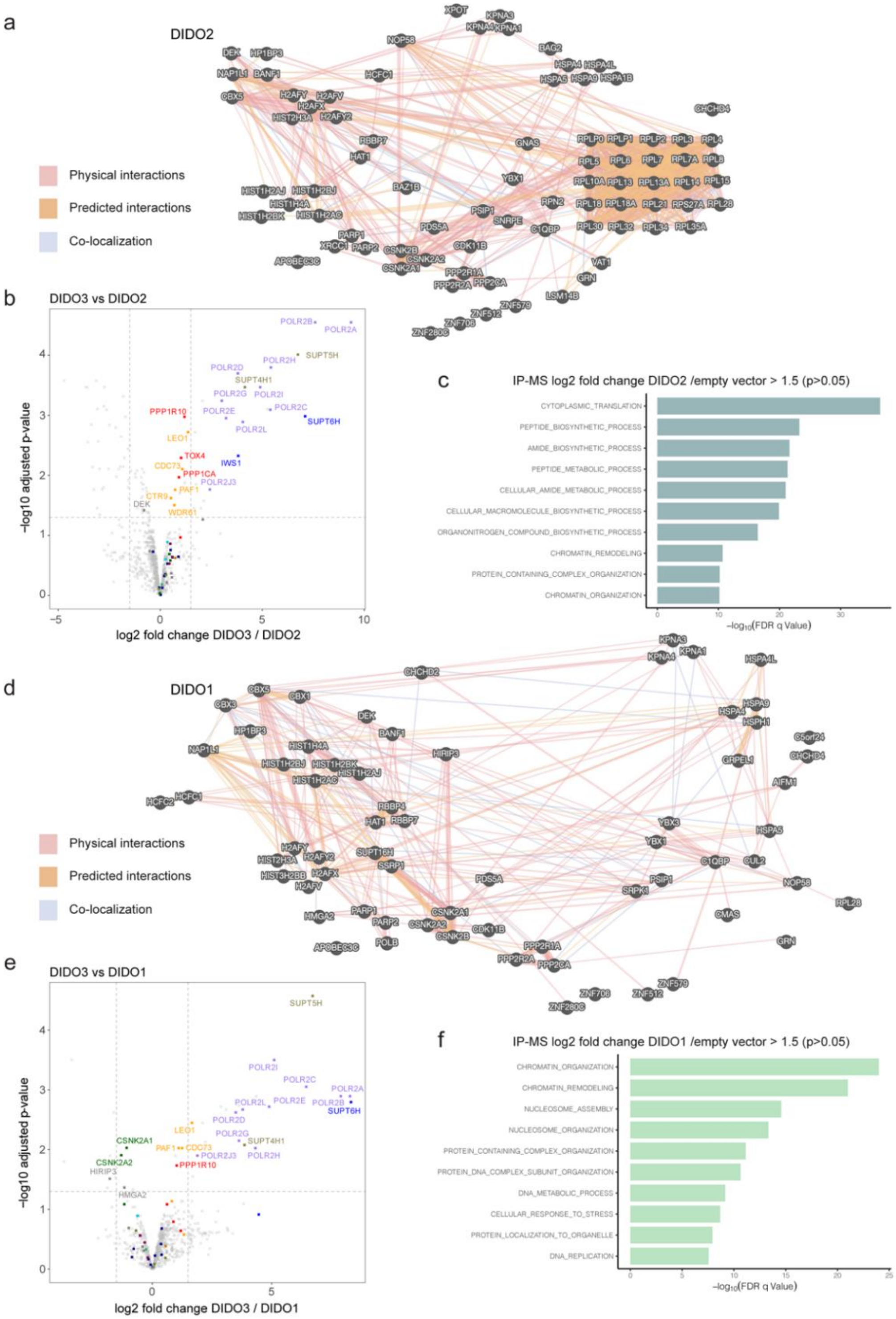

Supplementary Fig. 4: Analysis of DIDO2 and DIDO1 interactomes by mass spectrometry. a,d GeneMANIA interaction maps for **a** DIDO2 and **d** DIDO1. **b,e** Volcano plots for **b** DIDO3 vs DIDO2 and **e** DIDO3 vs DIDO1. The Pol II complex is indicated in purple, SPT6/IWS1 in blue, DSIF (SPT4/SPT5) in olive, PAF1C in orange, PP1 in red, CK2 in dark green, chromatin-associated factors in grey. The experiments were carried out in three individual replicates. Statistical calculations were performed using the LIMMA package in R[4]. Adjusted p-values were calculated using the Benjamini-Hochberg correction for multiple testing. Mass spectrometry data are provided in Supplementary Data 1. **c,f** GO analysis of interactomes of **c** DIDO1 and **f** DIDO2. GSEA biological processes tool was used[5].

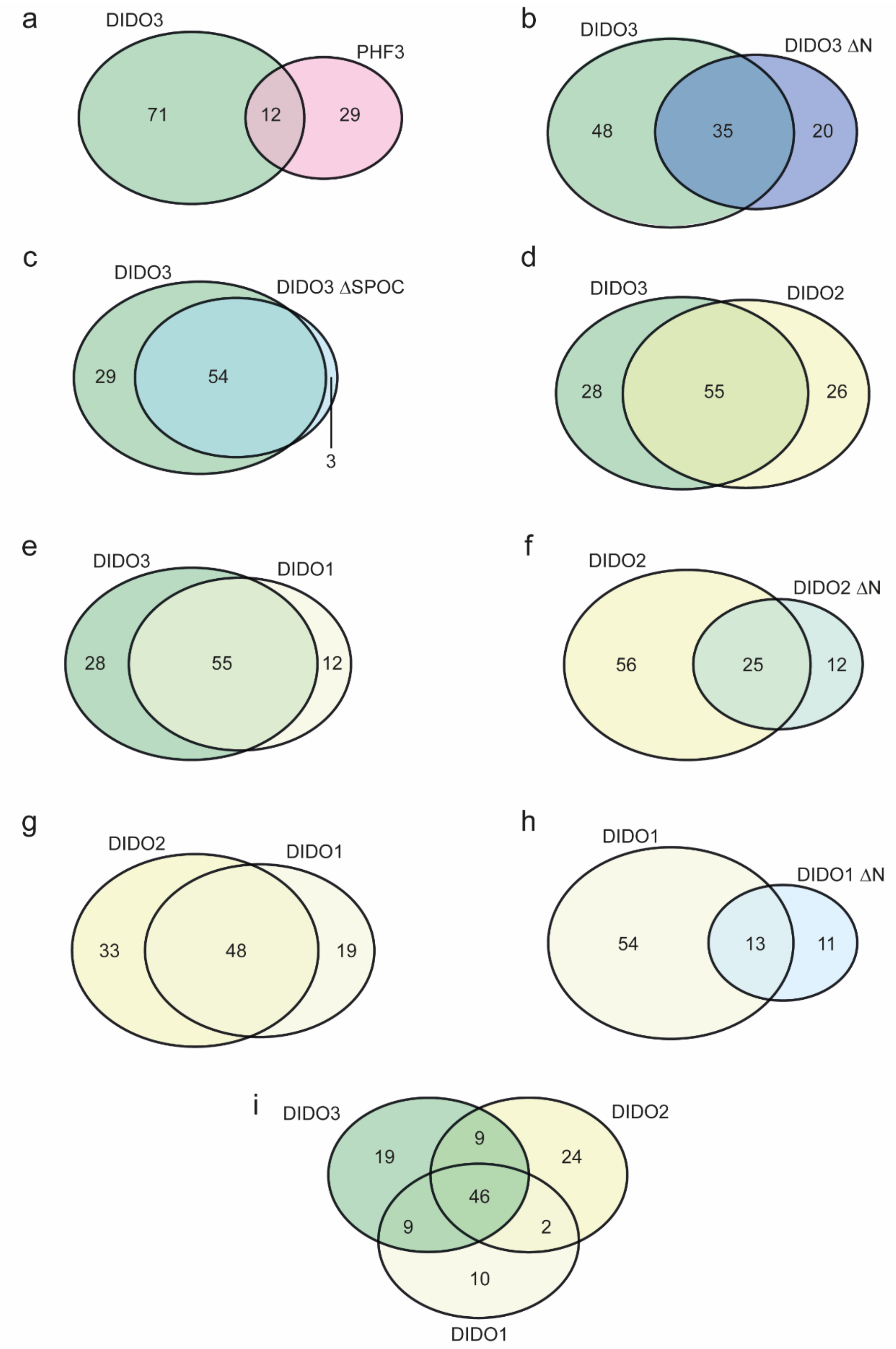

Supplementary Fig. 5: Venn diagrams showing overlaps between DIDO3 and PHF3 interactors as well as different DIDO constructs. Common and unique interactors are shown in Supplementary Data 1.

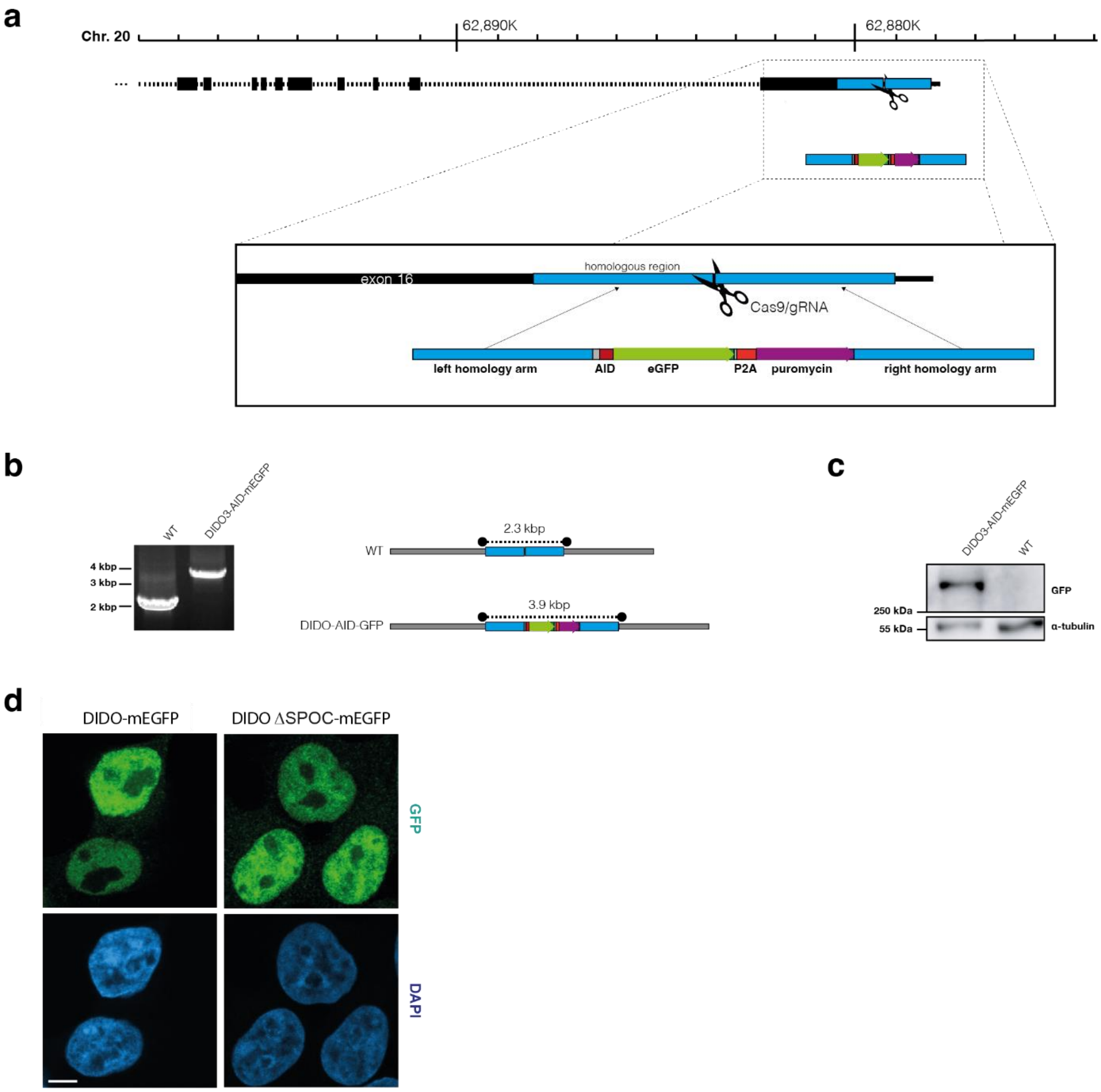

Supplementary Fig. 6: Generation of mEGFP-tagged DIDO cell lines. a Schematic overview of the CRISPR/Cas9 editing strategy. Endogenous DIDO3 was C-terminally tagged with mEGFP in WT, PHF3 KO, PHF3 ΔSPOC and DIDO ΔSPOC background. **b,c,d** Validation of DIDO-mEGFP tagging by **b** PCR, **c** Western blotting and **d** immunofluorescence microscopy using anti-GFP antibody (scale bar = 5 μm). The experiments were performed once. Source data are provided as a Source Data file.

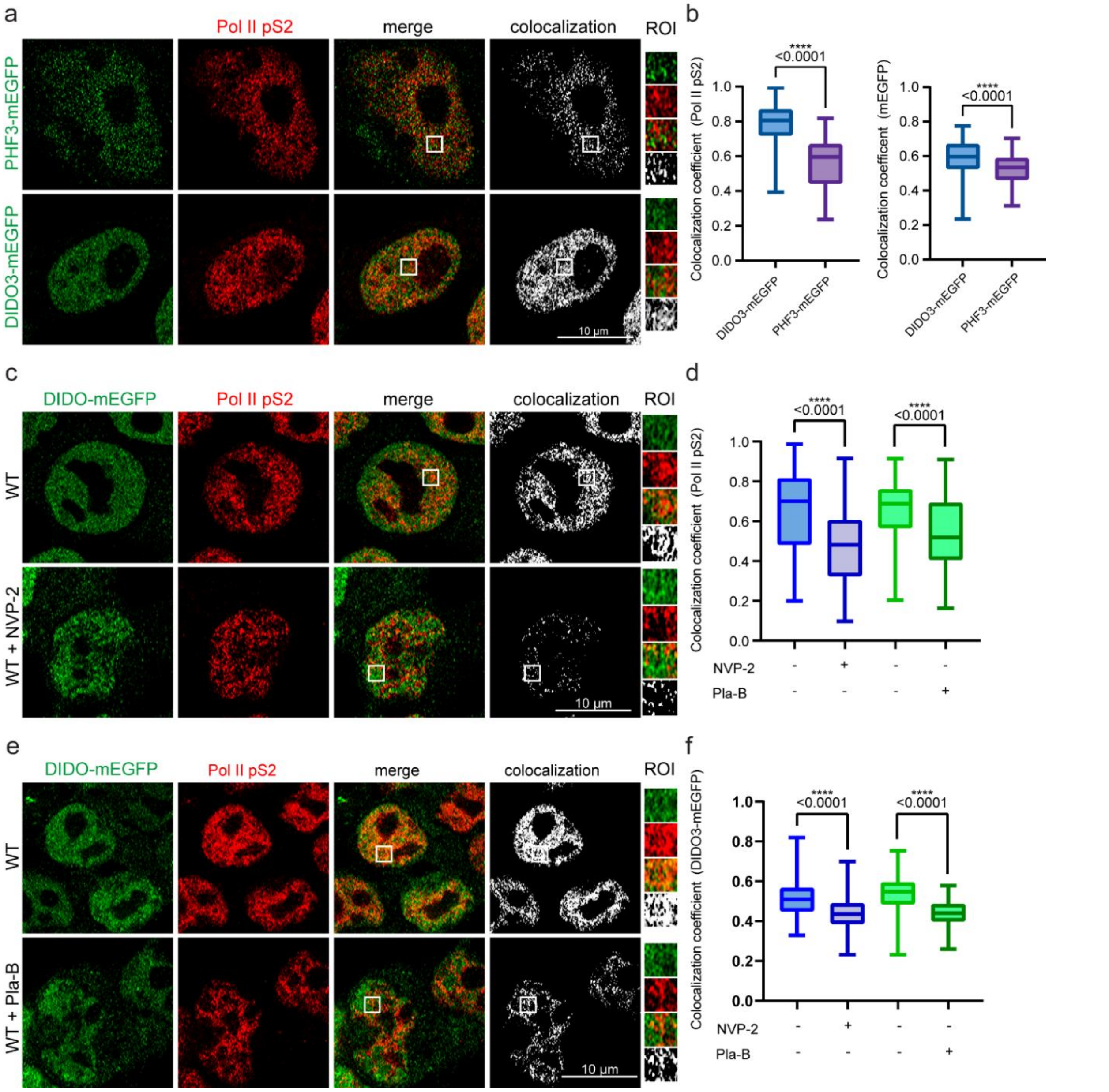

Supplementary Fig. 7: DIDO3 colocalizes with Pol II in transcription elongation and splicing dependent manner. a Representative Airyscan high resolution images of PHF3-mEGFP or DIDO3-mEGFP (IF staining with rabbit anti-GFP + Alexa Fluor 488, green) and Pol II pS2 (Alexa Fluor 594, red). Colocalization analysis of clusters that overlap in both channels (white). **b** Quantification of the fraction of Pol II pS2 colocalizing with DIDO3 or PHF3 (Manders coefficient 1; left panel) or fraction of DIDO3 or PHF3 colocalizing with Pol II pS2 (Manders coefficient 2; right panel) (N=146). Box and whiskers plot depicting the median (line), 25-75% interquartile range (box borders) and minimum/maximum (whiskers) are shown. Two-tailed unpaired Student's t-test with Welch's correction was used to determine statistical significance. **c,e** Representative Airyscan high resolution images of DIDO3-mEGFP (IF staining with rabbit anti-GFP + Alexa Fluor 488, green) and Pol II pS2 (Alexa Fluor 594, red) from untreated cells and **c** cells treated with 250 nM NVP-2 for 3 h or **e** cells treated with 1 µM Pla-B for 4 h. Colocalization analysis of clusters that overlap in both channels (white). Scale bar=10µm. **d,f** Quantification of the fraction of DIDO3 colocalizing with Pol II pS2 from **c,e** (N=175). Box and whiskers plot depicting the median (line), 25-75% interquartile range (box borders) and minimum/maximum (whiskers) are shown. Each experiment was repeated three times with comparable results. Two-tailed unpaired Student's t-test with Welch's correction was used to determine statistical significance. Source data are provided as a Source Data file.

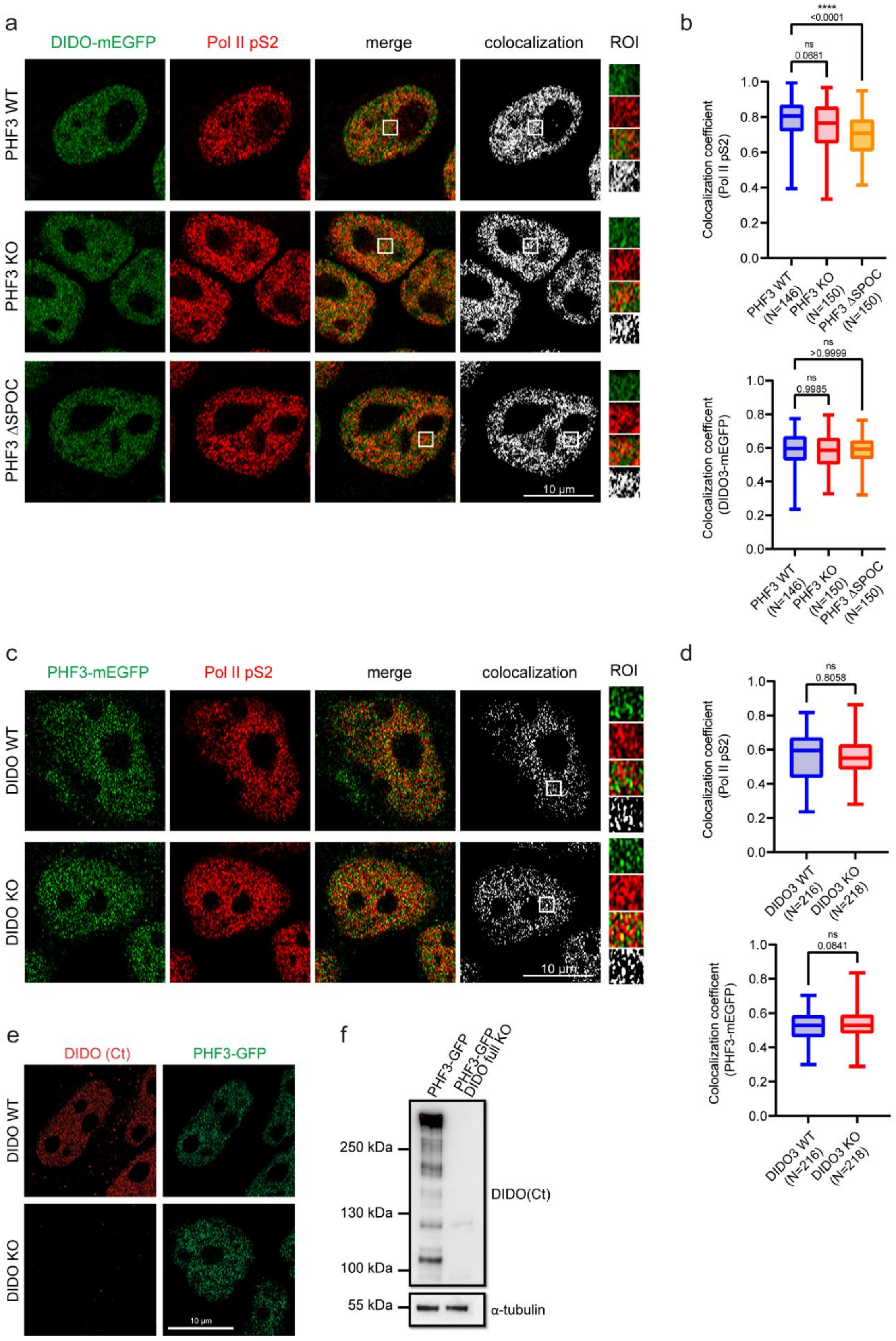

(Supplementary Fig. 8, see figure legend on the next page)

Supplementary Fig. 8: PHF3 is not required for DIDO3 colocalization with Pol II and vice versa. a Representative Airyscan high resolution images of DIDO3-mEGFP (IF staining with rabbit anti-GFP + Alexa Fluor 488, green) and Pol II pS2 (Alexa Fluor 594, red) in PHF3 WT, PHF3 KO and PHF3 ΔSPOC HEK293T cells. Colocalization analysis of clusters that overlap in both channels (white). **b** Quantification of the fraction of Pol II pS2 colocalizing with DIDO3 (Manders coefficient 1; top panel) or fraction of DIDO3 colocalizing with Pol II pS2 (Manders coefficient 2; bottom panel). Box and whiskers plot depicting the median (line), 25-75% interquartile range (box borders) and minimum/maximum (whiskers) are shown. Two-tailed unpaired Student's t-test with Welch's correction was used to determine statistical significance. **c** Representative Airyscan high resolution images of PHF3-mEGFP (IF staining with rabbit anti-GFP + Alexa Fluor 488, green) and Pol II pS2 (Alexa Fluor 594, red) in DIDO3 WT and DIDO3 KO HEK293T cells. Colocalization analysis of clusters that overlap in both channels (white). Scale bar=10µm. **d** Quantification of the fraction of Pol II pS2 colocalizing with PHF3 (Manders coefficient 1; top panel) or fraction of PHF3 colocalizing with Pol II pS2 (Manders coefficient 2; bottom panel). Box and whiskers plot depicting the median (line), 25-75% interquartile range (box borders) and minimum/maximum (whiskers) are shown. Each experiment was repeated three times with comparable results. One-way ANOVA with Brown-Forsythe and Welch's correction was used to determine statistical significance. **e** Airyscan high resolution images (63x) and **f** Western blot analysis of DIDO knockout with DIDO3 C-terminal antibody. Experiments were performed twice. Source data are provided as a Source Data file.

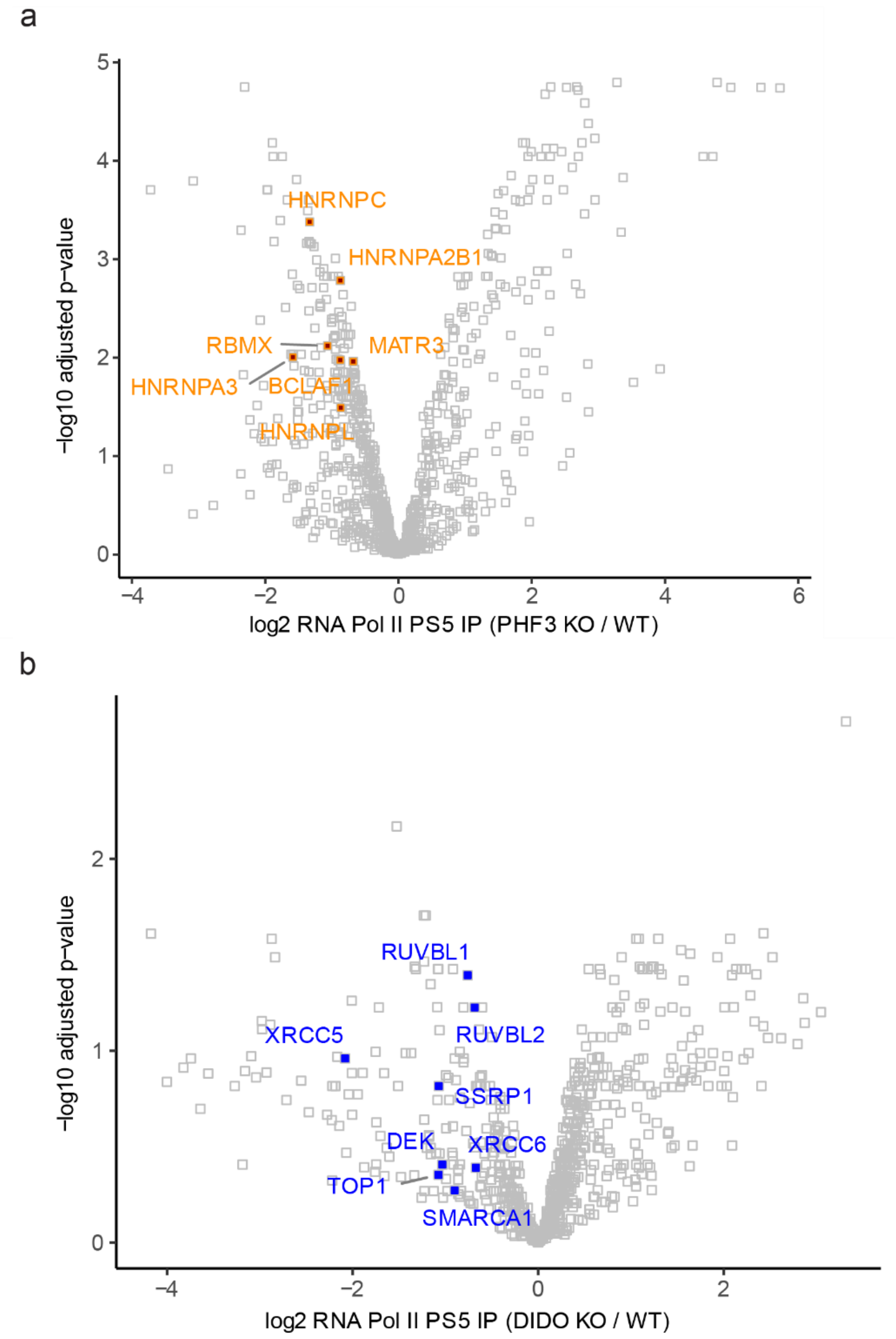

Supplementary Fig. 9: Mass spectrometry analysis of differential Pol II pS5 interactome in PHF3 KO and DIDO KO cells. Volcano plots for **a** PHF3 KO vs WT and **b** DIDO KO vs WT. Each experiment was repeated three times. Statistical calculations were performed using the LIMMA package in R[4]. Adjusted p-values were calculated using the Benjamini-Hochberg correction for multiple testing. Mass spectrometry data are provided in Supplementary Data 2 and 3.

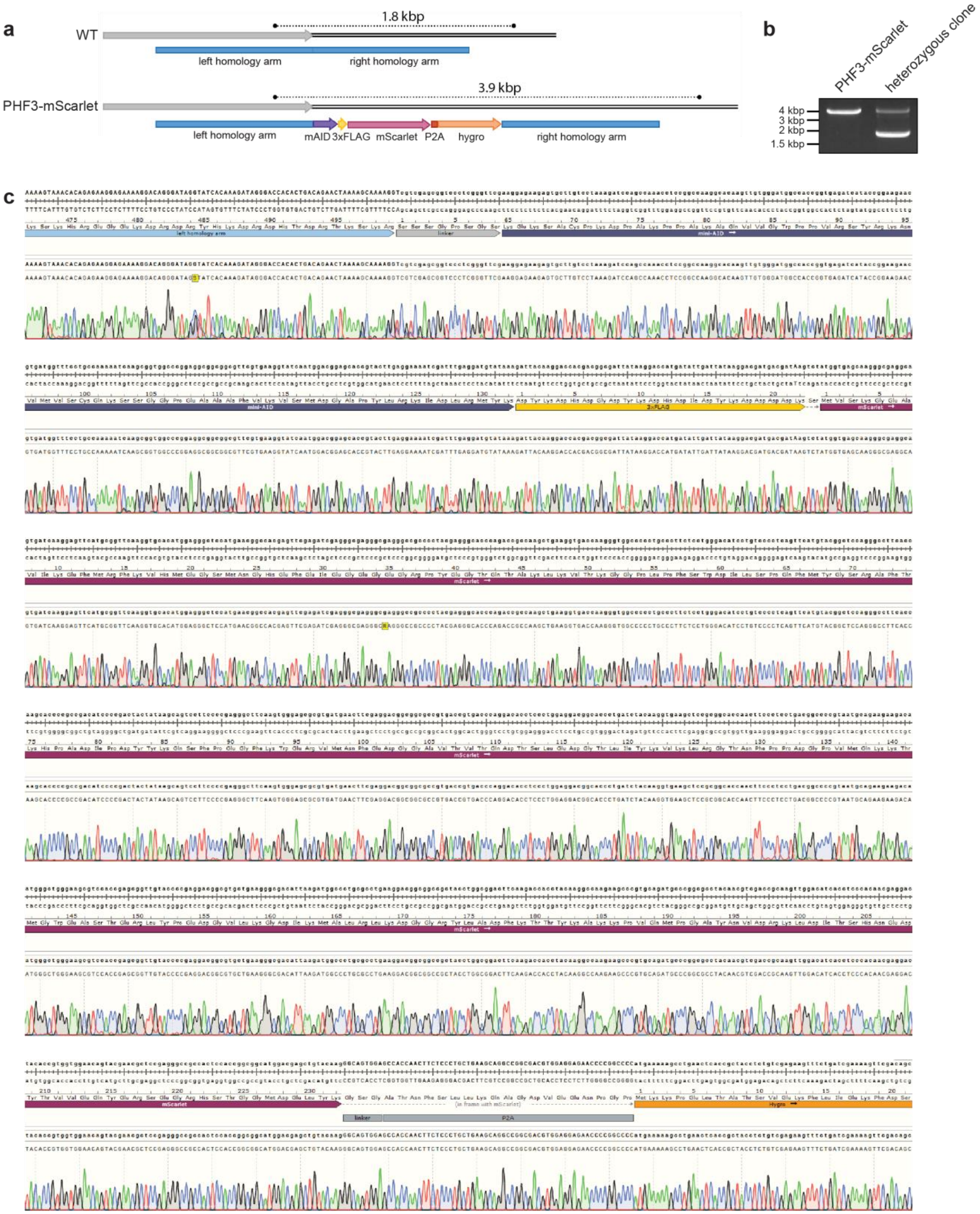

Supplementary Fig. 10: Generation of DIDO-mEGFP PHF3-mScarlet cell line. a A schematic overview of CRISPR/Cas9 strategy. PHF3 was endogenously tagged with mScarlet at the C-terminus in DIDO-mEGFP background. **b,c** Validation of endogenous tagging of PHF3 with mAID-3xFLAG-mScarlet by **b** PCR and **c** Sanger sequencing. The experiments were performed once. Source data are provided as a Source Data file.

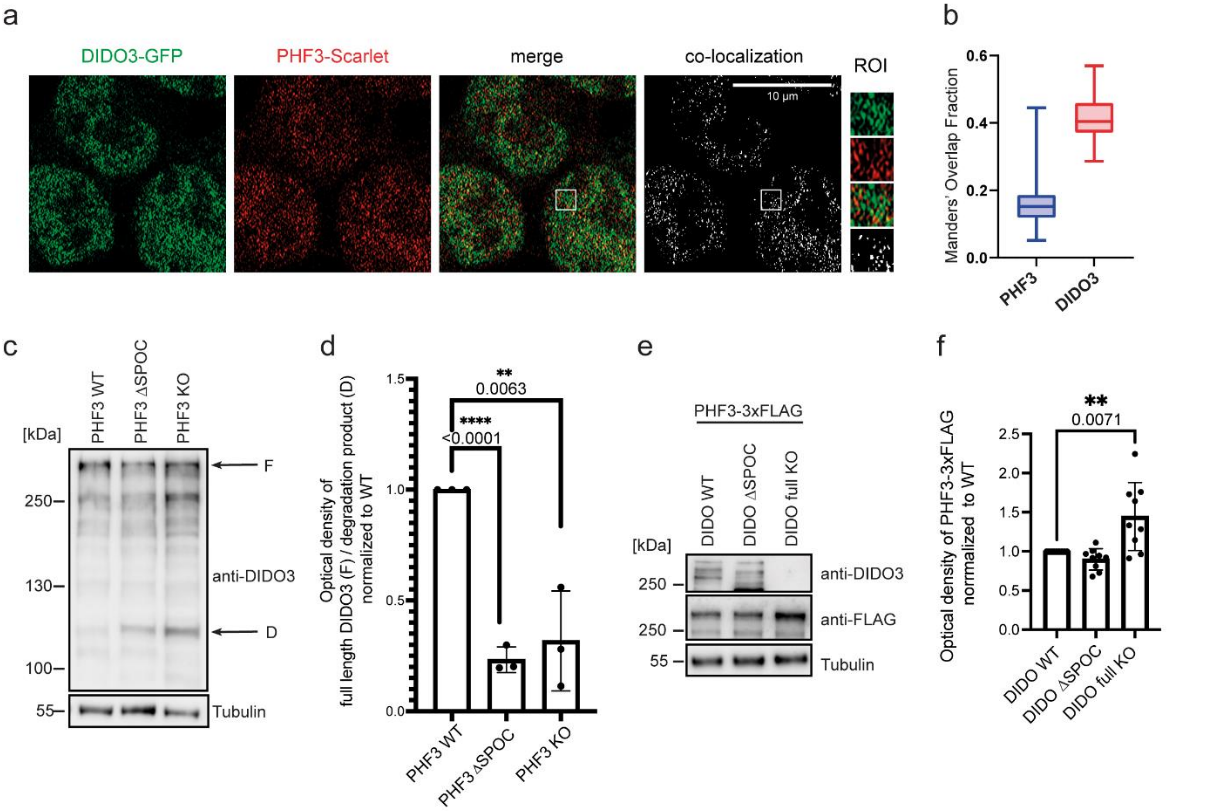

Supplementary Fig. 11: PHF3 and DIDO3 form a complex in cells and mutually affect protein stability. a High-resolution Airy scan images of DIDO3-mEGFP (green) and PHF3-3xFLAG-mScarlet (red) in HEK293T cells. Anti-GFP and anti-FLAG antibodies were used to enhance endogenous signals. Co-localization analysis of clusters that overlap in both channels (white). Scale bar=10µm. Experiments were performed twice with comparable results. **b** Quantification of the fraction of DIDO3 co-localizing with PHF3 indicated by Manders' overlap coefficient (MOC) (N=171). Box and whiskers plot depicting the median (line), 25-75% interquartile range (box borders) and minimum/maximum (whiskers) are shown. **c** Western blot showing DIDO3 levels in PHF3 WT, PHF3 ΔSPOC and PHF3 KO (N=3). Full length DIDO3 and its degradation product are indicated with an arrow. **d** Quantification of the relative optical density of the full length DIDO3 band / the degradation product normalized to WT. Data are presented as mean values ± standard deviation. One-tailed, two-sample equal variance t-test was used to determine p-values. **e** Western blot showing PHF3 levels in DIDO3 WT, DIDO3 ΔSPOC and DIDO3 KO (N=9). PHF3 was endogenously tagged with 3xFLAG at the C-terminus to facilitate detection. **f** Quantification of the relative optical density of PHF3 normalized to WT. Data are presented as mean values ± standard deviation. One-tailed, two-sample equal variance t-test was used to determine p-values. Source data are provided as a Source Data file.

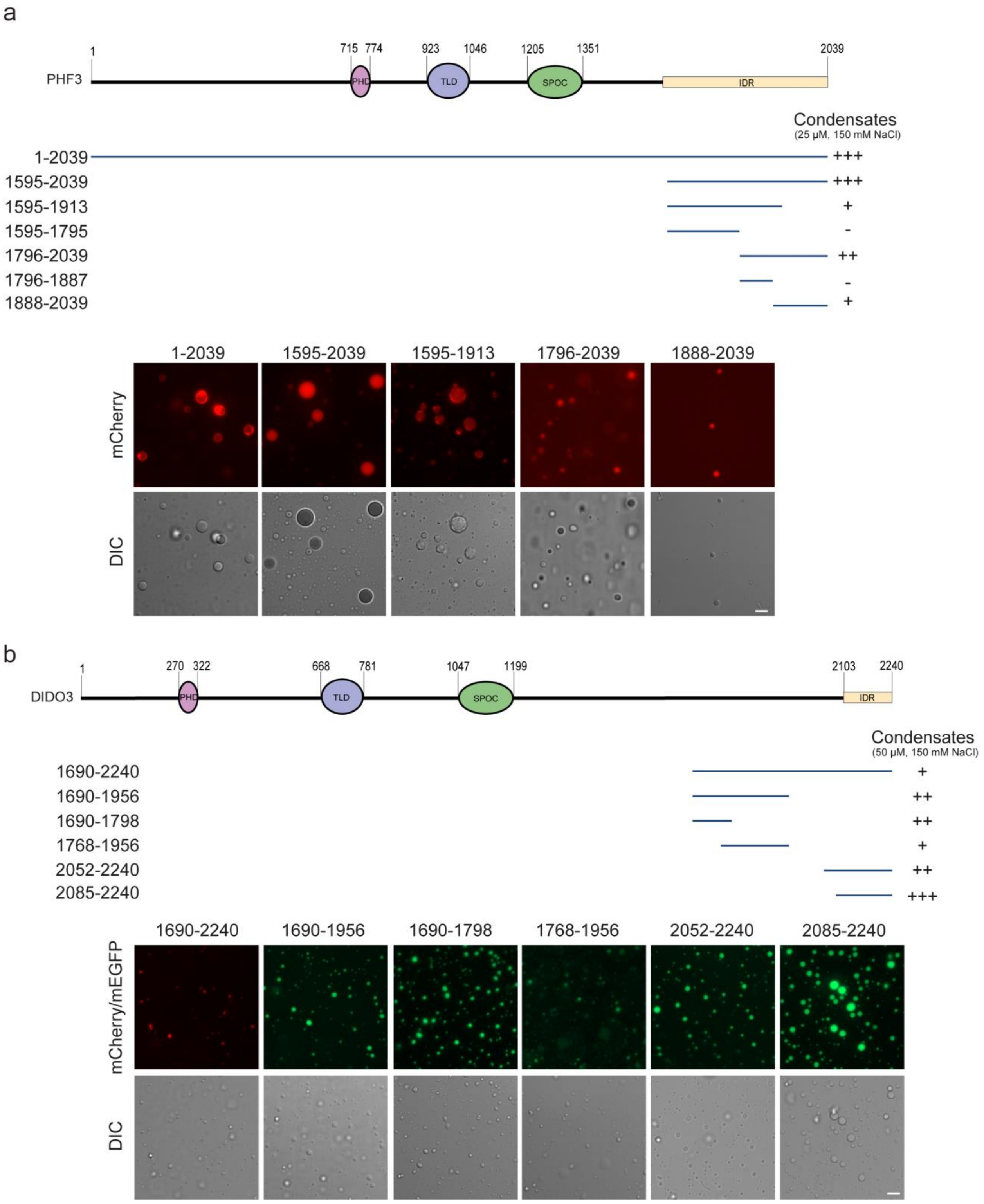

Supplementary Fig. 12: PHF3 and DIDO3 C-terminal regions form condensates *in vitro*. a PHF3 (25 µM) and **b** DIDO3 (50 µM) C-terminal constructs were tested for condensate formation *in vitro* in the presence of 150 mM NaCl and 10% dextran. Representative images are shown. Scale bar=5µm. The experiments were performed in two independent replicates.

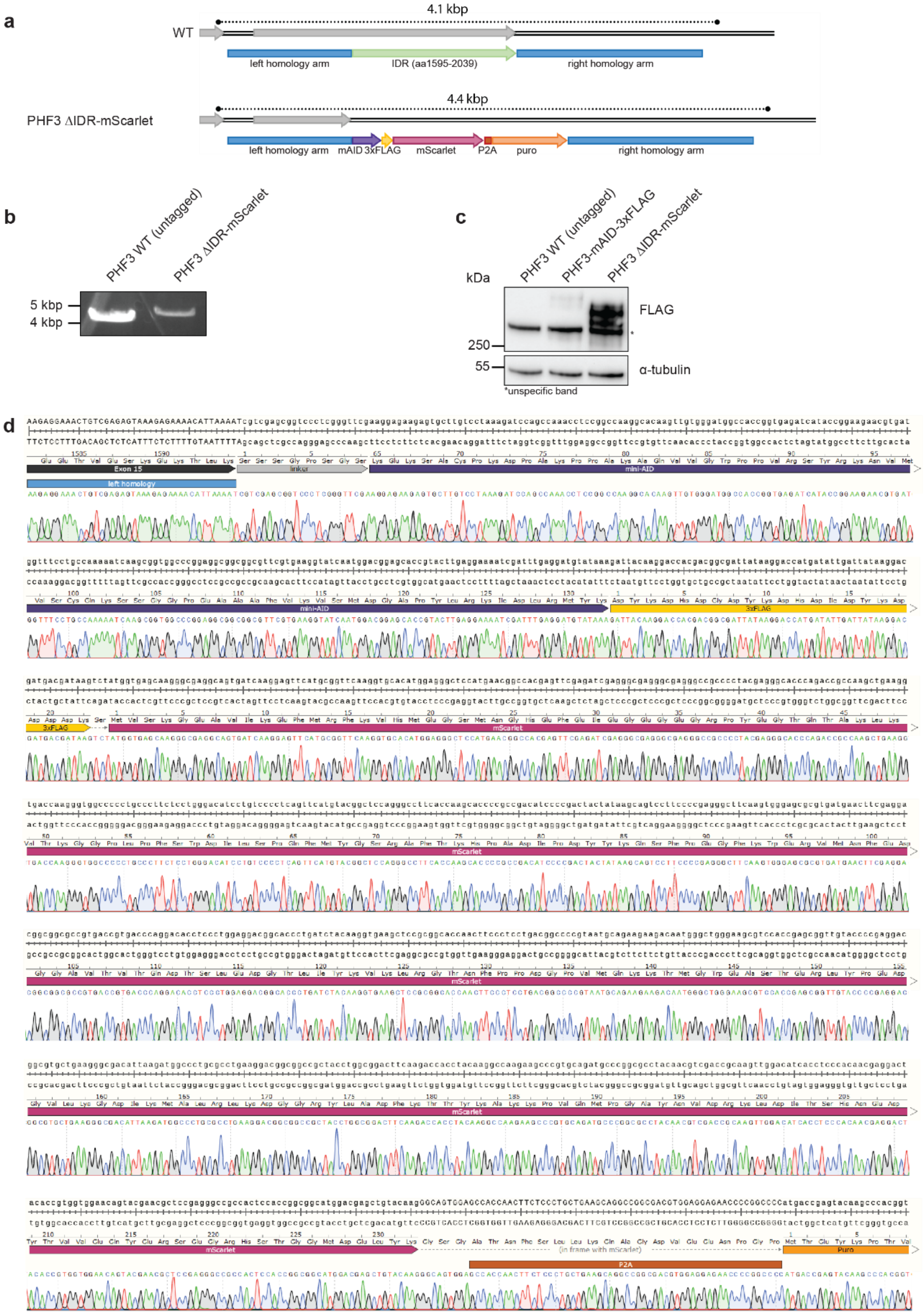

Supplementary Fig. 13: Generation of PHF3 ΔIDR-mScarlet cell line. a A schematic overview of CRISPR/Cas9 strategy. **b,c,d** Validation of endogenous C-terminal truncation and tagging of PHF3 with mAID-3xFLAG-mScarlet by **b** PCR, **c** Western blotting and **d** Sanger sequencing. The experiments were performed once. Source data are provided as a Source Data file.

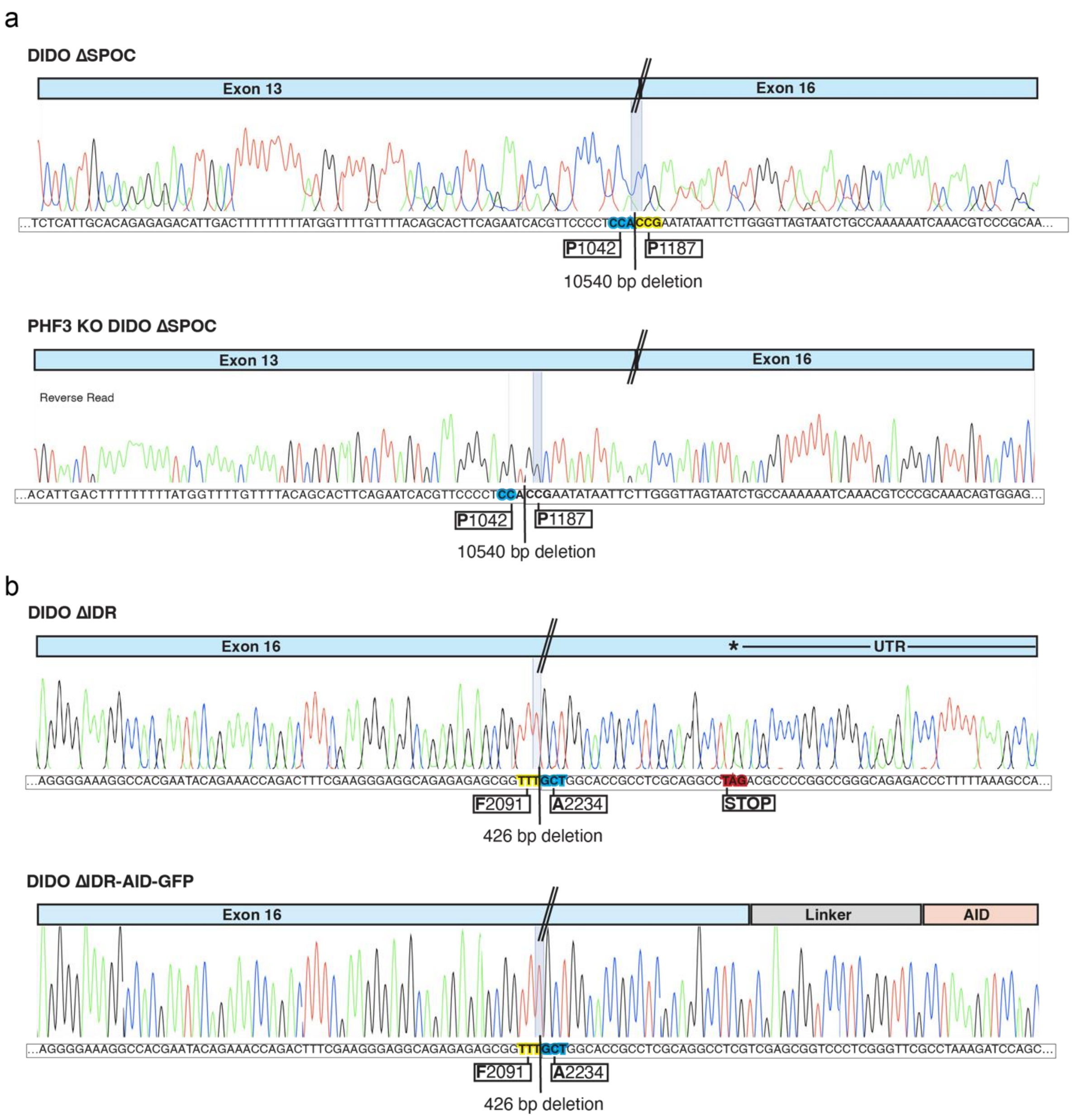

Supplementary Fig. 14: Sequencing results confirm deletion of the SPOC and the IDR domain by Non-Homologous End-Joining (NHEJ). a Sanger sequencing confirms the deletion of 10540 bp in WT and PHF3 KO HEK293T cells. Deletion spans from exon 13 to exon 16 containing the sequence coding for the DIDO SPOC domain. **b** Sanger sequencing shows the deletion of 426 bp containing the sequence for IDR in WT and DIDO-GFP HEK293T cells.

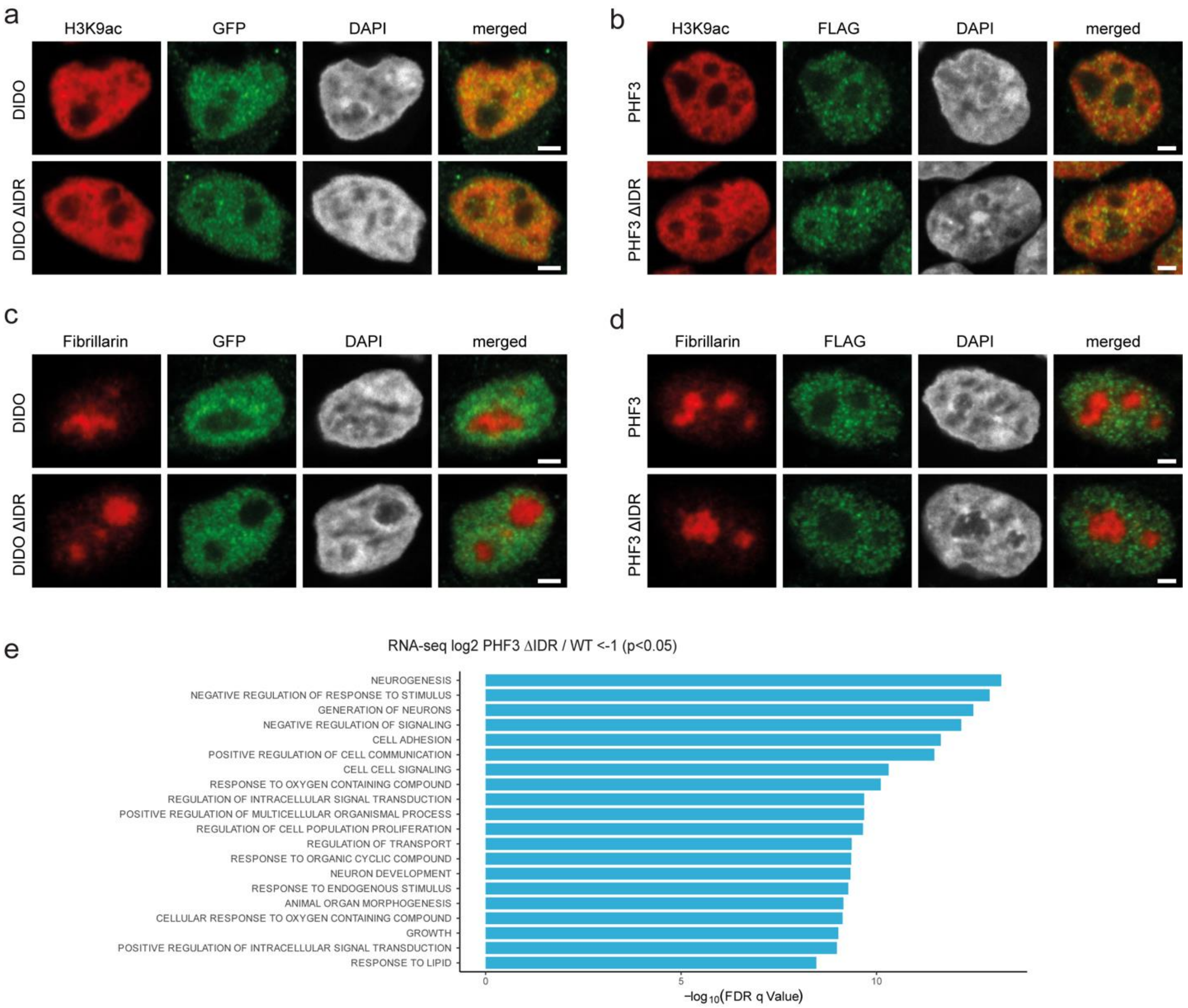

Supplementary Fig. 15: DIDO3 and PHF3 localize in euchromatic regions and are excluded from nucleoli. Immunofluorescence images showing **a,b** H3K9ac and **c,d** fibrillarin in **a,c** mEGFP-tagged DIDO3 and DIDO ΔIDR, and **b,d** PHF3-mScarlet and PHF3ΔIDR-mScarlet. **c,d** Scale bar=2µm. Experiments were performed twice, representative images are shown. **e** GO analysis of genes downregulated in PHF3 ΔIDR cells according to RNA-seq. GSEA Biological processes tool was used[5].

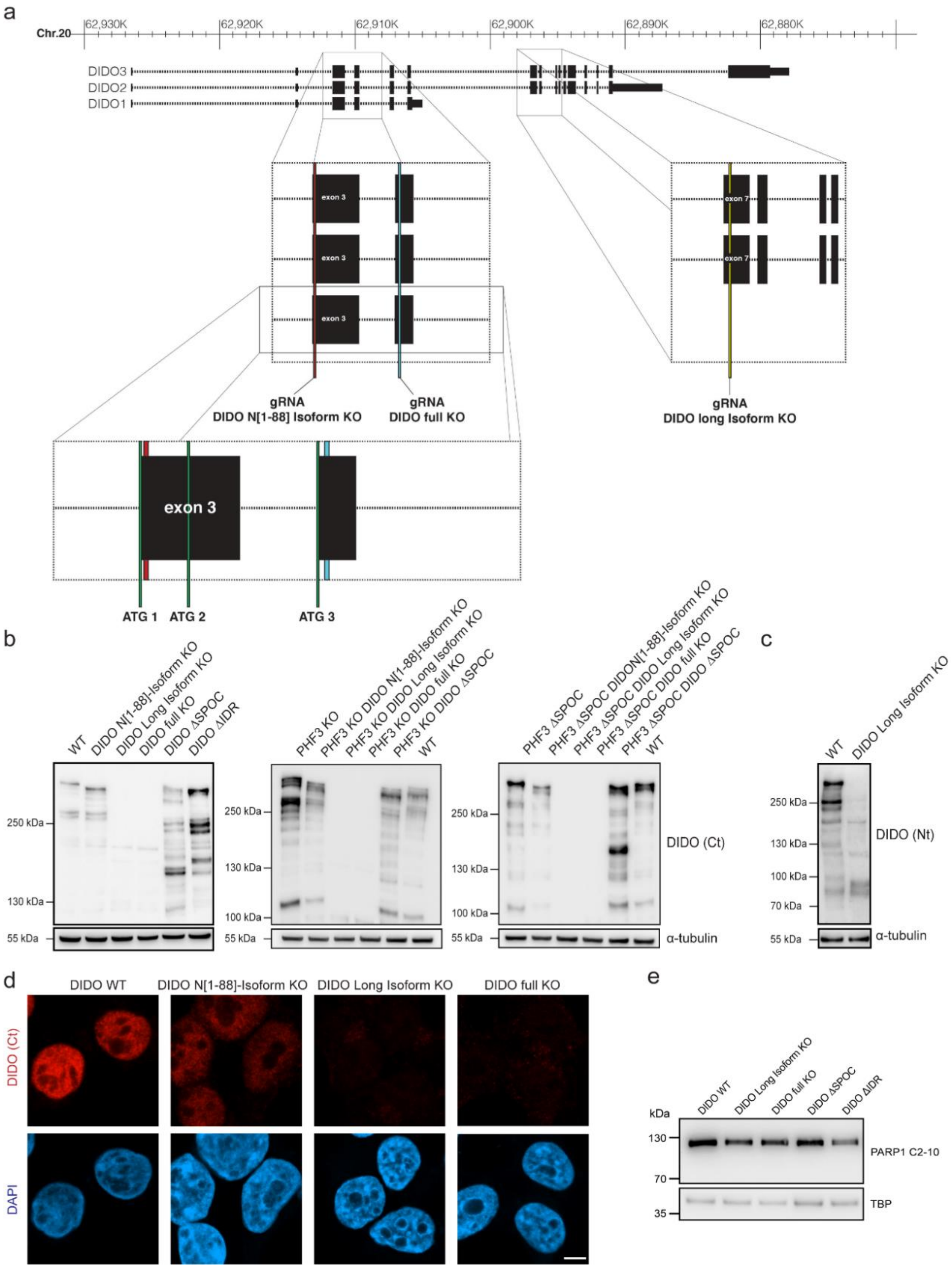

Supplementary Fig. 16: CRISPR/Cas9 toolset to study DIDO3 function. **a** gRNA target regions for the generation of DIDO knock-outs. Red region is the target for DIDO N[1-88]-Isoform KO, blue region for DIDO full KO, yellow region for DIDO Long Isoform KO. Translational start codons (ATG1-3) are indicated in green. **b,c** Western blot and **d** immunofluorescence analysis of DIDO3 expression in knock-out and domain deletion cell lines using an antibody against DIDO3 C-terminus (b,d) or N-terminus (c). Scale bar=5 µm. **e** Western blot analysis of PARP1 (116 kDa) and cleaved PARP1 (85 kDa). TBP was used as a loading control. The experiments in **b-e** were perfomed once. Source data are provided as a Source Data file.

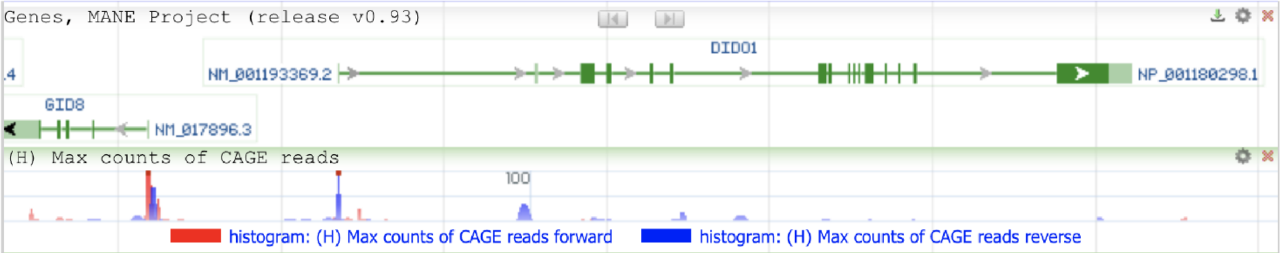

Supplementary Fig. 17: The *DIDO1* gene has three transcription start sites. Maximum counts of FANTOM 5 CAGE reads are shown in a screenshot from NCBI. Tracks show three peaks upstream of exon 3. For the *DIDO1* gene the reverse reads are relevant (blue).

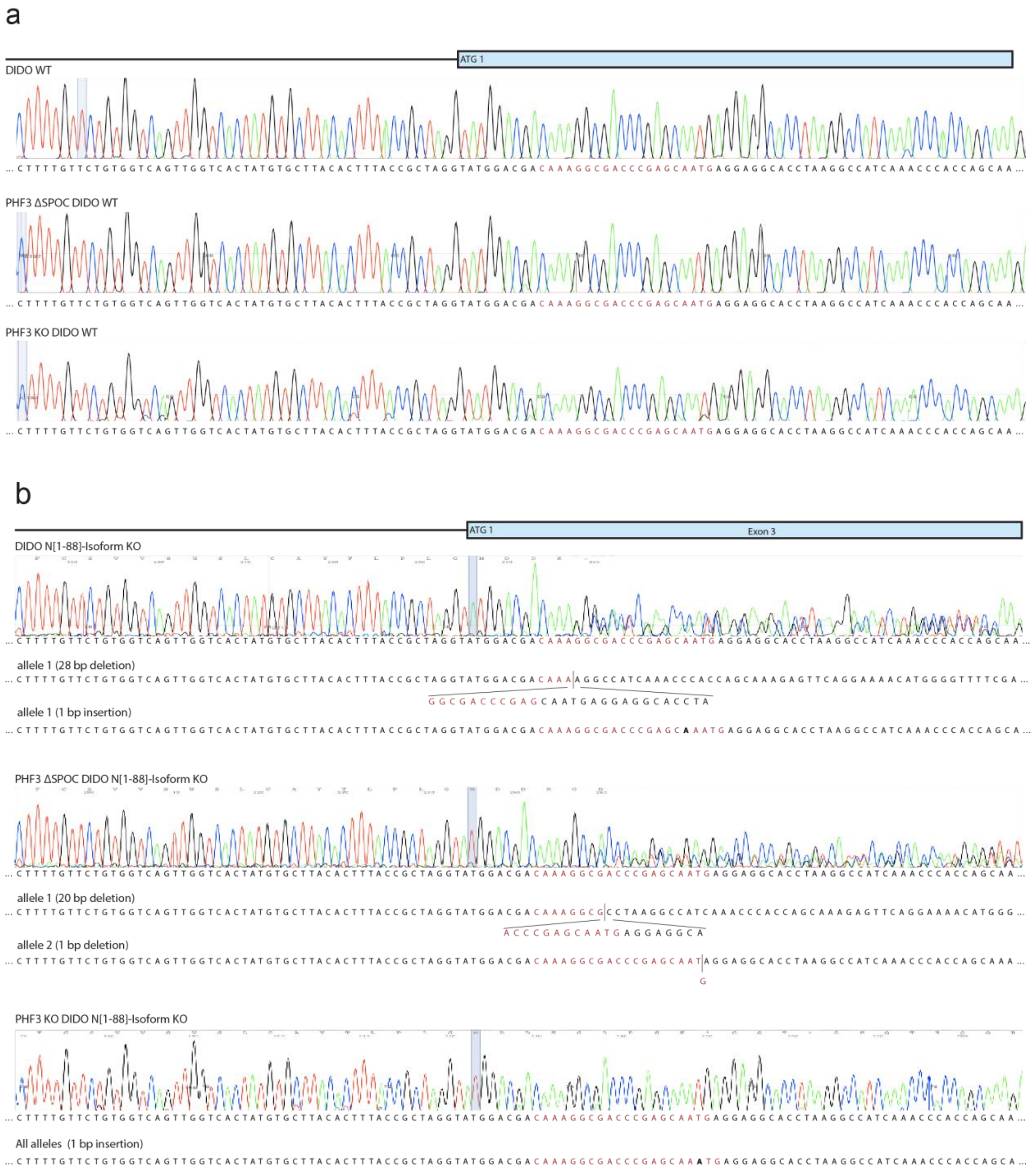
a
ATG 1
DIDO WT
...CTTTTGTTCTGTGGTCAGTTGGTCACTATGTGCTTACACTTTACCGCTAGGTATGGACGACAAAGGCGACCCGAGCAATGAGGAGGCACCTAAGGCCATCAAACCCACCAGCAA...
PHF3 ΔSPOC DIDO WT
...CTTTTGTTCTGTGGTCAGTTGGTCACTATGTGCTTACACTTTACCGCTAGGTATGGACGACAAAGGCGACCCGAGCAATGAGGAGGCACCTAAGGCCATCAAACCCACCAGCAA...
PHF3 KO DIDO WT
...CTTTTGTTCTGTGGTCAGTTGGTCACTATGTGCTTACACTTTACCGCTAGGTATGGACGACAAAGGCGACCCGAGCAATGAGGAGGCACCTAAGGCCATCAAACCCACCAGCAA...
b
ATG 1
Exon 3
DIDO N[1-88]-Isoform KO
...CTTTTGTTCTGTGGTCAGTTGGTCACTATGTGCTTACACTTTACCGCTAGGTATGGACGACAAAGGCGACCCGAGCAATGAGGAGGCACCTAAGGCCATCAAACCCACCAGCAA...
allele 1 (28 bp deletion)
...CTTTTGTTCTGTGGTCAGTTGGTCACTATGTGCTTACACTTTACCGCTAGGTATGGACGACAAAAGGCCATCAAACCCACCAGCAAAGAGTTCAGGAAAACATGGGGTTTTCGA...
GGCGACCCGAGCAATGAGGAGGCACCTA
allele 1 (1 bp insertion)
...CTTTTGTTCTGTGGTCAGTTGGTCACTATGTGCTTACACTTTACCGCTAGGTATGGACGACAAAGGCGACCCGAGCAAATGAGGAGGCACCTAAGGCCATCAAACCCACCAGCA...
PHF3 ΔSPOC DIDO N[1-88]-Isoform KO
...CTTTTGTTCTGTGGTCAGTTGGTCACTATGTGCTTACACTTTACCGCTAGGTATGGACGACAAAGGCGACCCGAGCAATGAGGAGGCACCTAAGGCCATCAAACCCACCAGCAA...
allele 1 (20 bp deletion)
...CTTTTGTTCTGTGGTCAGTTGGTCACTATGTGCTTACACTTTACCGCTAGGTATGGACGACAAAGGCGCCTAAGGCCATCAAACCCACCAGCAAAGAGTTCAGGAAAACATGGG...
ACCCGAGCAATGAGGAGGCA
allele 2 (1 bp deletion)
...CTTTTGTTCTGTGGTCAGTTGGTCACTATGTGCTTACACTTTACCGCTAGGTATGGACGACAAAGGCGACCCGAGCAATAGGAGGCACCTAAGGCCATCAAACCCACCAGCAAA...
G
PHF3 KO DIDO N[1-88]-Isoform KO
...CTTTTGTTCTGTGGTCAGTTGGTCACTATGTGCTTACACTTTACCGCTAGGTATGGACGACAAAGGCGACCCGAGCAATGAGGAGGCACCTAAGGCCATCAAACCCACCAGCAA...
All alleles (1 bp insertion)
...CTTTTGTTCTGTGGTCAGTTGGTCACTATGTGCTTACACTTTACCGCTAGGTATGGACGACAAAGGCGACCCGAGCAAATGAGGAGGCACCTAAGGCCATCAAACCCACCAGCA...

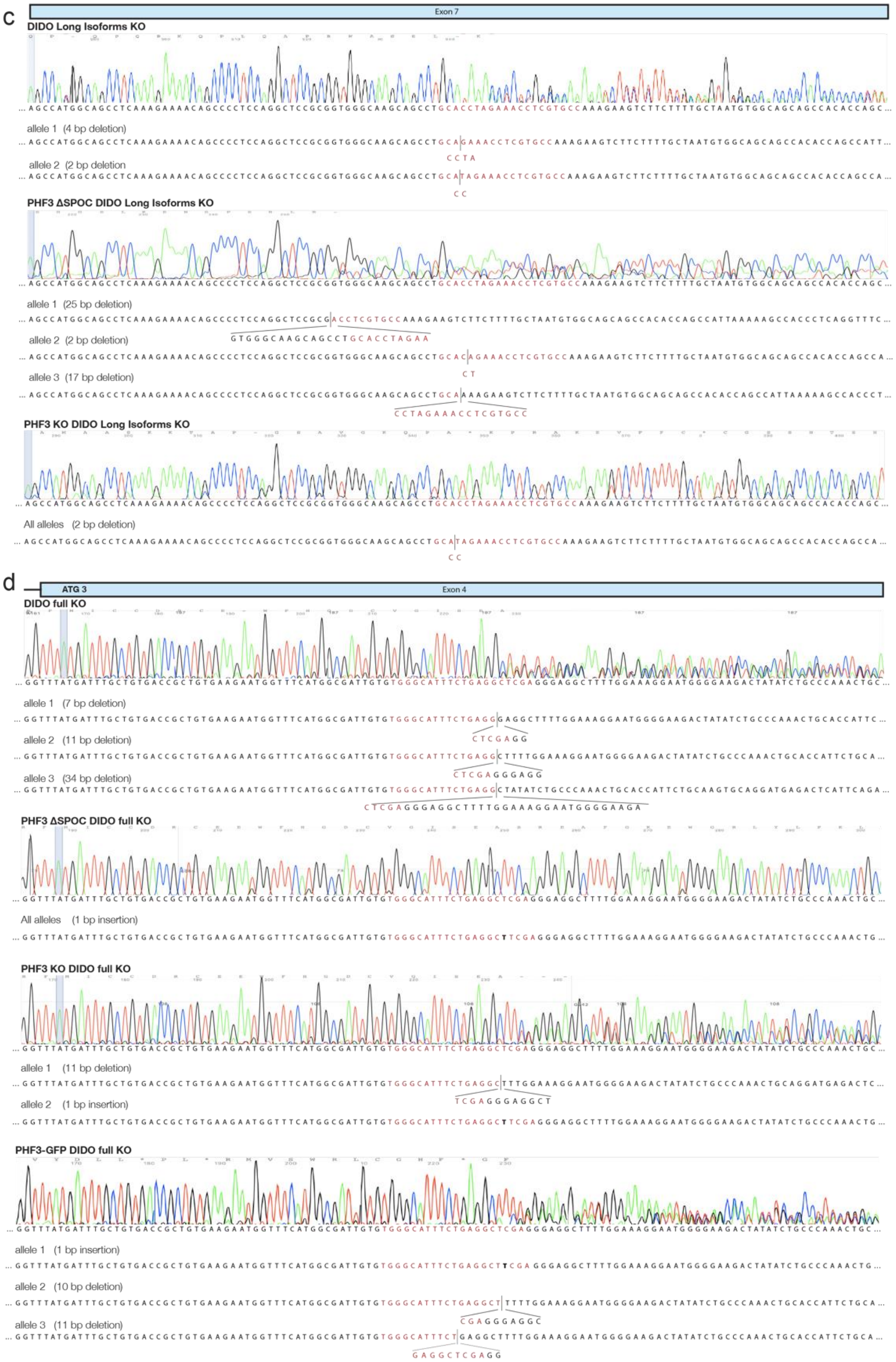

Supplementary Fig. 18: Validation of DIDO knock-out cell lines by Sanger sequencing. a WT cell lines show no deviation from the wild-type sequence. **b** DIDO N[1-88]-Isoform KO, **c** DIDO Long Isoform KO, and **d** DIDO full KO in WT, PHF3 ΔSPOC, PHF3 KO and PHF3-GFP HEK293T cells. All alleles show a frameshift either through insertion or deletion of base pairs. Source data are provided as a Source Data file.

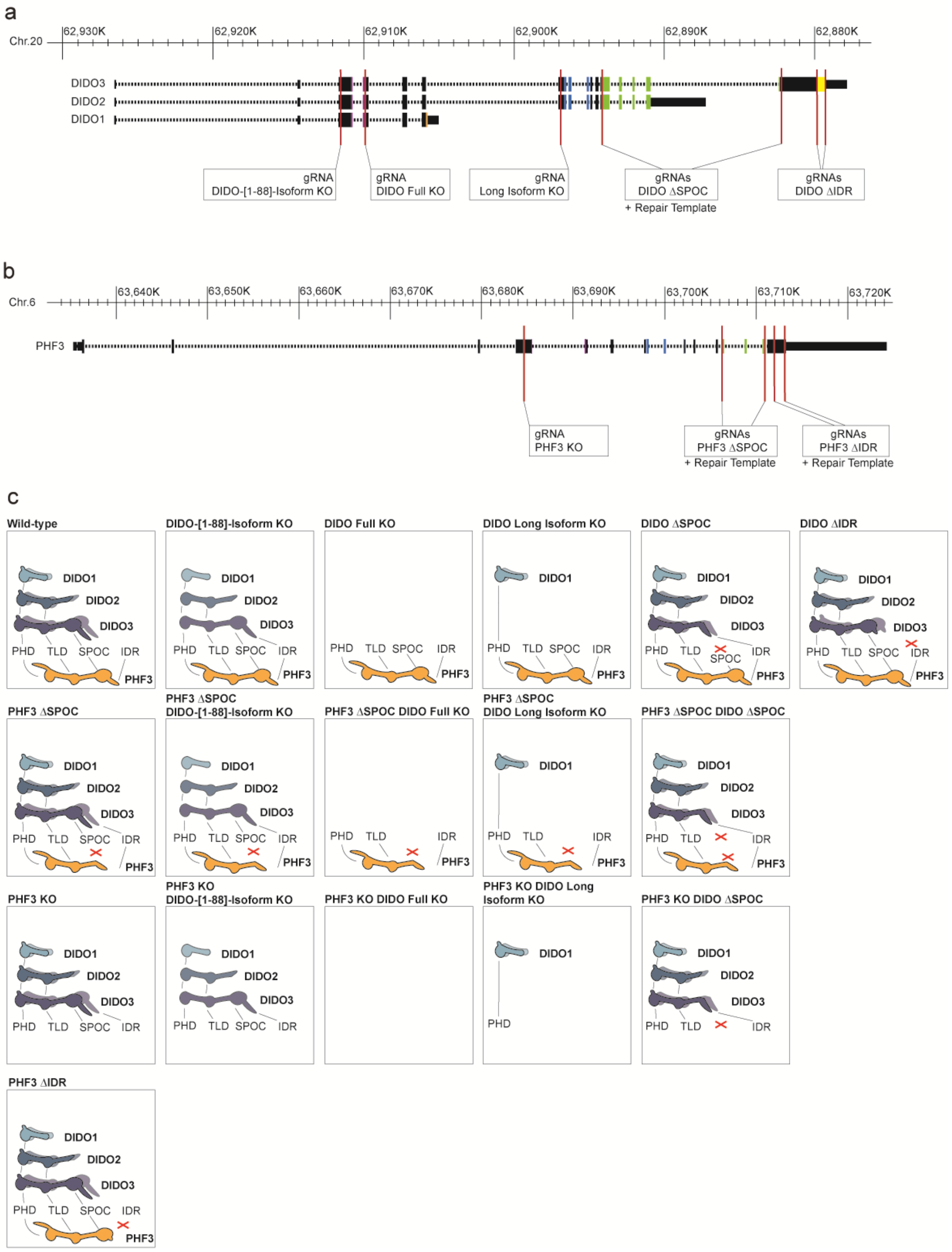

Supplementary Fig. 19: A schematic overview of cell lines used in this study. a A schematic overview of gRNAs used to generate DIDO mutants. **b** A schematic overview of gRNAs used to generate PHF3 mutants. **c** A schematic overview of PHF3 and DIDO isoforms present in each cell line used in this study. Each of the three DIDO isoforms exists as a full length version and a truncated version lacking the first 88 amino acids. DIDO-[1-88]-Isoform KO cell lines lack the full length versions, while the truncated versions are retained. In Full KO cell lines both versions of all isoforms have been eliminated.

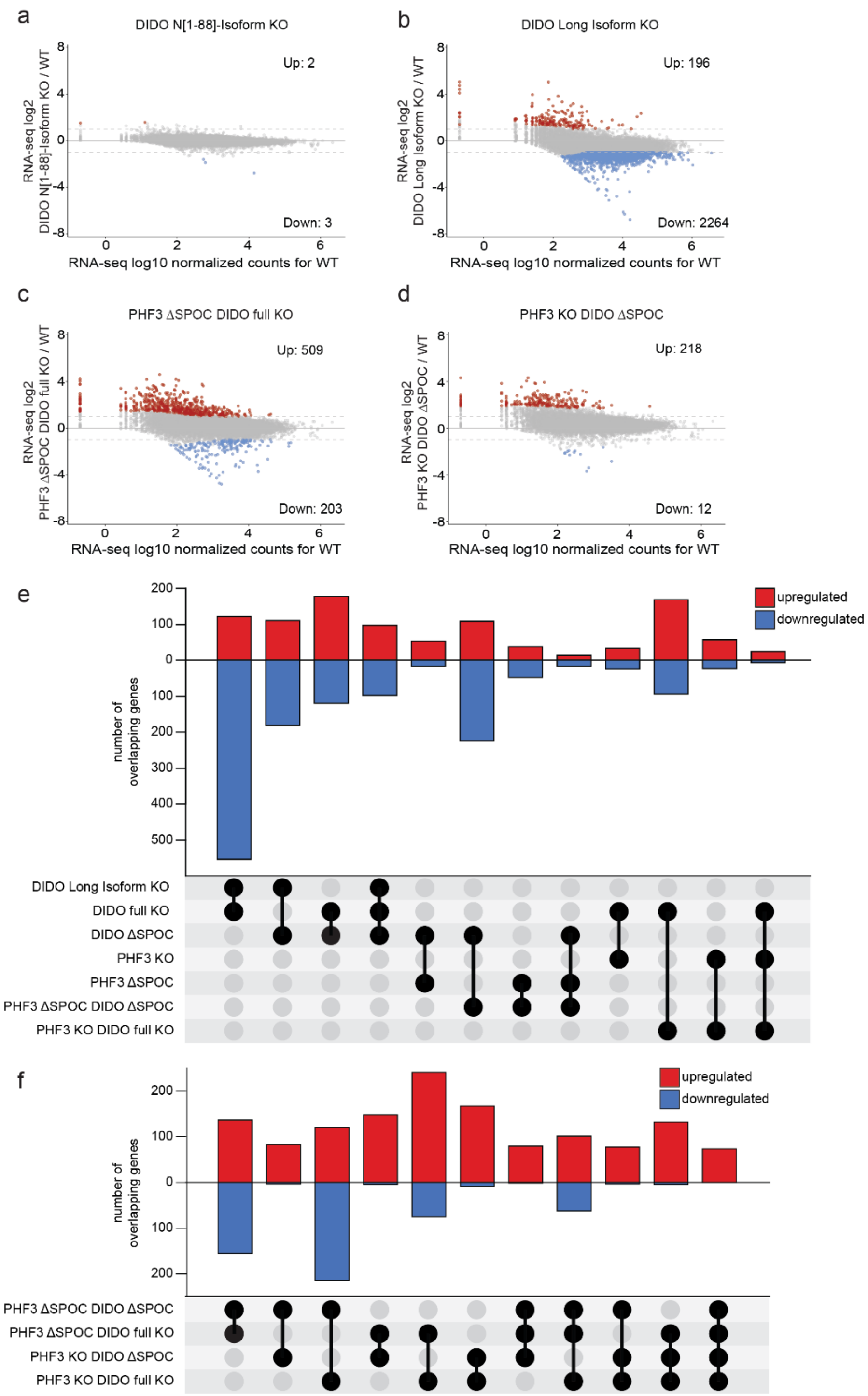

Supplementary Fig. 20: RNA-seq analysis of single and combined DIDO and PHF3 KO and ΔSPOC HEK293T cell lines.a-d MA plots showing RNA-seq log2 fold change (mutant/WT) versus log10 mean expression in WT for **a** DIDO N[1-88]-Isoform KO, **b** DIDO Long Isoform KO, **c** PHF3 ΔSPOC DIDO full KO, **d** PHF3 KO DIDO ΔSPOC. Red and blue dots indicate upregulated and downregulated genes respectively with fold-change>2, p<0.05. The experiments were performed in three independent replicates. Statistical analysis was performed using Wald test as implemented in DESeq2[6]. Drosophila S2 cells were used for spike-in normalization. **e,f** UpSet plots showing the overlap of deregulated genes (fold change >2, p<0.05) between different genotypes. Upregulated genes are shown in red, downregulated genes in blue. Source data are provided as a Source Data file.

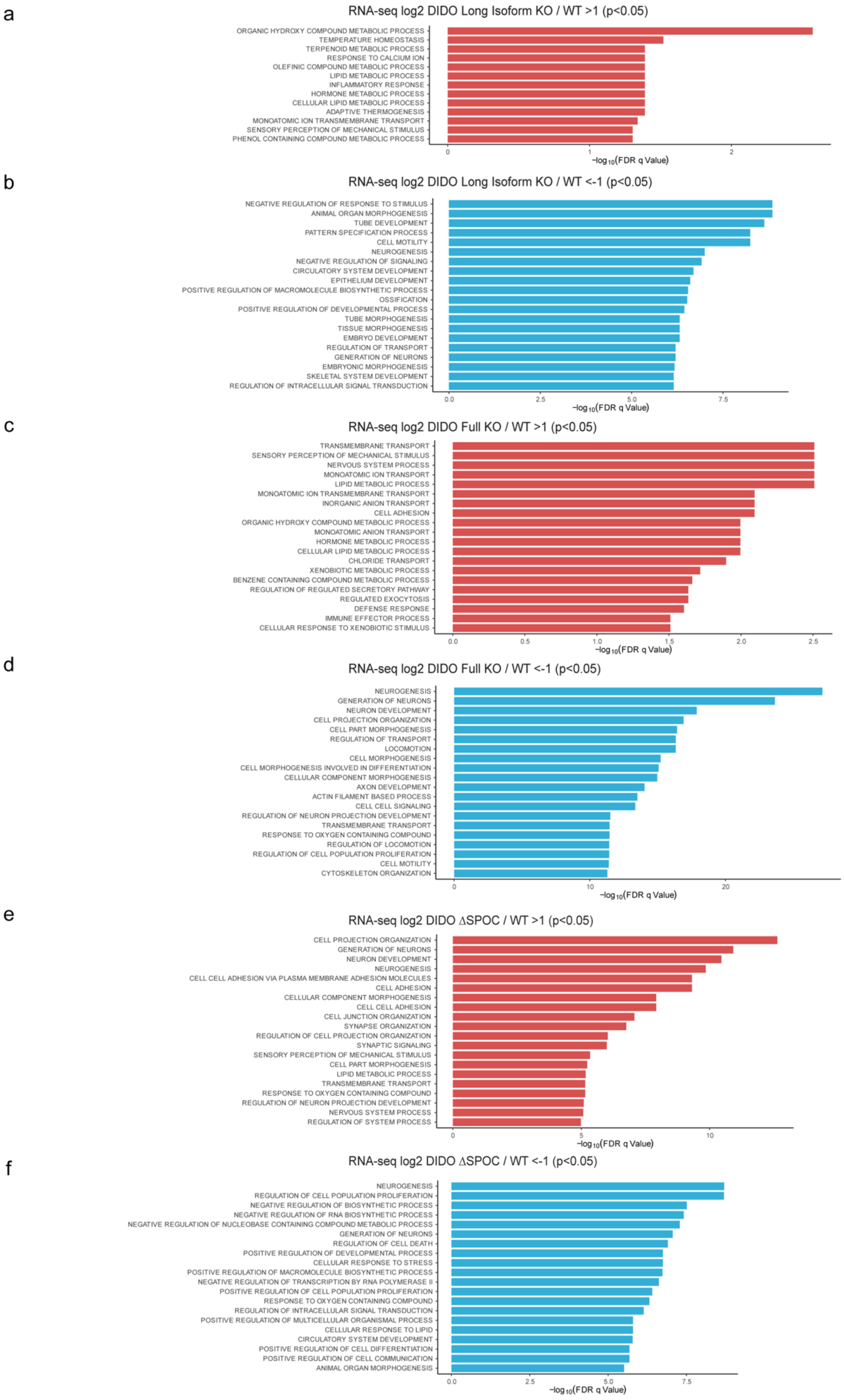

a
RNA-seq log2 DIDO Long Isoform KO / WT >1 (p<0.05)
ORGANIC HYDROXY COMPOUND METABOLIC PROCESS
TEMPERATURE HOMEOSTASIS
TERPENOID METABOLIC PROCESS
RESPONSE TO CALCIUM ION
OLEFINIC COMPOUND METABOLIC PROCESS
LIPID METABOLIC PROCESS
INFLAMMATORY RESPONSE
HORMONE METABOLIC PROCESS
CELLULAR LIPID METABOLIC PROCESS
ADAPTIVE THERMOGENESIS
MONOATOMIC ION TRANSMEMBRANE TRANSPORT
SENSORY PERCEPTION OF MECHANICAL STIMULUS
PHENOL CONTAINING COMPOUND METABOLIC PROCESS
0
1
2
−log10(FDR q Value)
b
RNA-seq log2 DIDO Long Isoform KO / WT <-1 (p<0.05)
NEGATIVE REGULATION OF RESPONSE TO STIMULUS
ANIMAL ORGAN MORPHOGENESIS
TUBE DEVELOPMENT
PATTERN SPECIFICATION PROCESS
CELL MOTILITY
NEUROGENESIS
NEGATIVE REGULATION OF SIGNALING
CIRCULATORY SYSTEM DEVELOPMENT
EPITHELIUM DEVELOPMENT
POSITIVE REGULATION OF MACROMOLECULE BIOSYNTHETIC PROCESS
OSSIFICATION
POSITIVE REGULATION OF DEVELOPMENTAL PROCESS
TUBE MORPHOGENESIS
TISSUE MORPHOGENESIS
EMBRYO DEVELOPMENT
REGULATION OF TRANSPORT
GENERATION OF NEURONS
EMBRYONIC MORPHOGENESIS
SKELETAL SYSTEM DEVELOPMENT
REGULATION OF INTRACELLULAR SIGNAL TRANSDUCTION
0.0
2.5
5.0
7.5
−log10(FDR q Value)
c
RNA-seq log2 DIDO Full KO / WT >1 (p<0.05)
TRANSMEMBRANE TRANSPORT
SENSORY PERCEPTION OF MECHANICAL STIMULUS
NERVOUS SYSTEM PROCESS
MONOATOMIC ION TRANSPORT
LIPID METABOLIC PROCESS
MONOATOMIC ION TRANSMEMBRANE TRANSPORT
INORGANIC ANION TRANSPORT
CELL ADHESION
ORGANIC HYDROXY COMPOUND METABOLIC PROCESS
MONOATOMIC ANION TRANSPORT
HORMONE METABOLIC PROCESS
CELLULAR LIPID METABOLIC PROCESS
CHLORIDE TRANSPORT
XENOBIOTIC METABOLIC PROCESS
BENZENE CONTAINING COMPOUND METABOLIC PROCESS
REGULATION OF REGULATED SECRETORY PATHWAY
REGULATED EXOCYTOSIS
DEFENSE RESPONSE
IMMUNE EFFECTOR PROCESS
CELLULAR RESPONSE TO XENOBIOTIC STIMULUS
0.0
0.5
1.0
1.5
2.0
2.5
−log10(FDR q Value)
d
RNA-seq log2 DIDO Full KO / WT <-1 (p<0.05)
NEUROGENESIS
GENERATION OF NEURONS
NEURON DEVELOPMENT
CELL PROJECTION ORGANIZATION
CELL PART MORPHOGENESIS
REGULATION OF TRANSPORT
LOCOMOTION
CELL MORPHOGENESIS
CELL MORPHOGENESIS INVOLVED IN DIFFERENTIATION
CELLULAR COMPONENT MORPHOGENESIS
AXON DEVELOPMENT
ACTIN FILAMENT BASED PROCESS
CELL CELL SIGNALING
REGULATION OF NEURON PROJECTION DEVELOPMENT
TRANSMEMBRANE TRANSPORT
RESPONSE TO OXYGEN CONTAINING COMPOUND
REGULATION OF LOCOMOTION
REGULATION OF CELL POPULATION PROLIFERATION
CELL MOTILITY
CYTOSKELETON ORGANIZATION
0
10
20
−log10(FDR q Value)
e
RNA-seq log2 DIDO ΔSPOC / WT >1 (p<0.05)
CELL PROJECTION ORGANIZATION
GENERATION OF NEURONS
NEURON DEVELOPMENT
NEUROGENESIS
CELL CELL ADHESION VIA PLASMA MEMBRANE ADHESION MOLECULES
CELL ADHESION
CELLULAR COMPONENT MORPHOGENESIS
CELL CELL ADHESION
CELL JUNCTION ORGANIZATION
SYNAPSE ORGANIZATION
REGULATION OF CELL PROJECTION ORGANIZATION
SYNAPTIC SIGNALING
SENSORY PERCEPTION OF MECHANICAL STIMULUS
CELL PART MORPHOGENESIS
LIPID METABOLIC PROCESS
TRANSMEMBRANE TRANSPORT
RESPONSE TO OXYGEN CONTAINING COMPOUND
REGULATION OF NEURON PROJECTION DEVELOPMENT
NERVOUS SYSTEM PROCESS
REGULATION OF SYSTEM PROCESS
0
5
10
−log10(FDR q Value)
f
RNA-seq log2 DIDO ΔSPOC / WT <-1 (p<0.05)
NEUROGENESIS
REGULATION OF CELL POPULATION PROLIFERATION
NEGATIVE REGULATION OF BIOSYNTHETIC PROCESS
NEGATIVE REGULATION OF RNA BIOSYNTHETIC PROCESS
NEGATIVE REGULATION OF NUCLEOBASE CONTAINING COMPOUND METABOLIC PROCESS
GENERATION OF NEURONS
REGULATION OF CELL DEATH
POSITIVE REGULATION OF DEVELOPMENTAL PROCESS
CELLULAR RESPONSE TO STRESS
POSITIVE REGULATION OF MACROMOLECULE BIOSYNTHETIC PROCESS
NEGATIVE REGULATION OF TRANSCRIPTION BY RNA POLYMERASE II
POSITIVE REGULATION OF CELL POPULATION PROLIFERATION
RESPONSE TO OXYGEN CONTAINING COMPOUND
REGULATION OF INTRACELLULAR SIGNAL TRANSDUCTION
POSITIVE REGULATION OF MULTICELLULAR ORGANISMAL PROCESS
CELLULAR RESPONSE TO LIPID
CIRCULATORY SYSTEM DEVELOPMENT
POSITIVE REGULATION OF CELL DIFFERENTIATION
POSITIVE REGULATION OF CELL COMMUNICATION
ANIMAL ORGAN MORPHOGENESIS
0.0
2.5
5.0
7.5
−log10(FDR q Value)

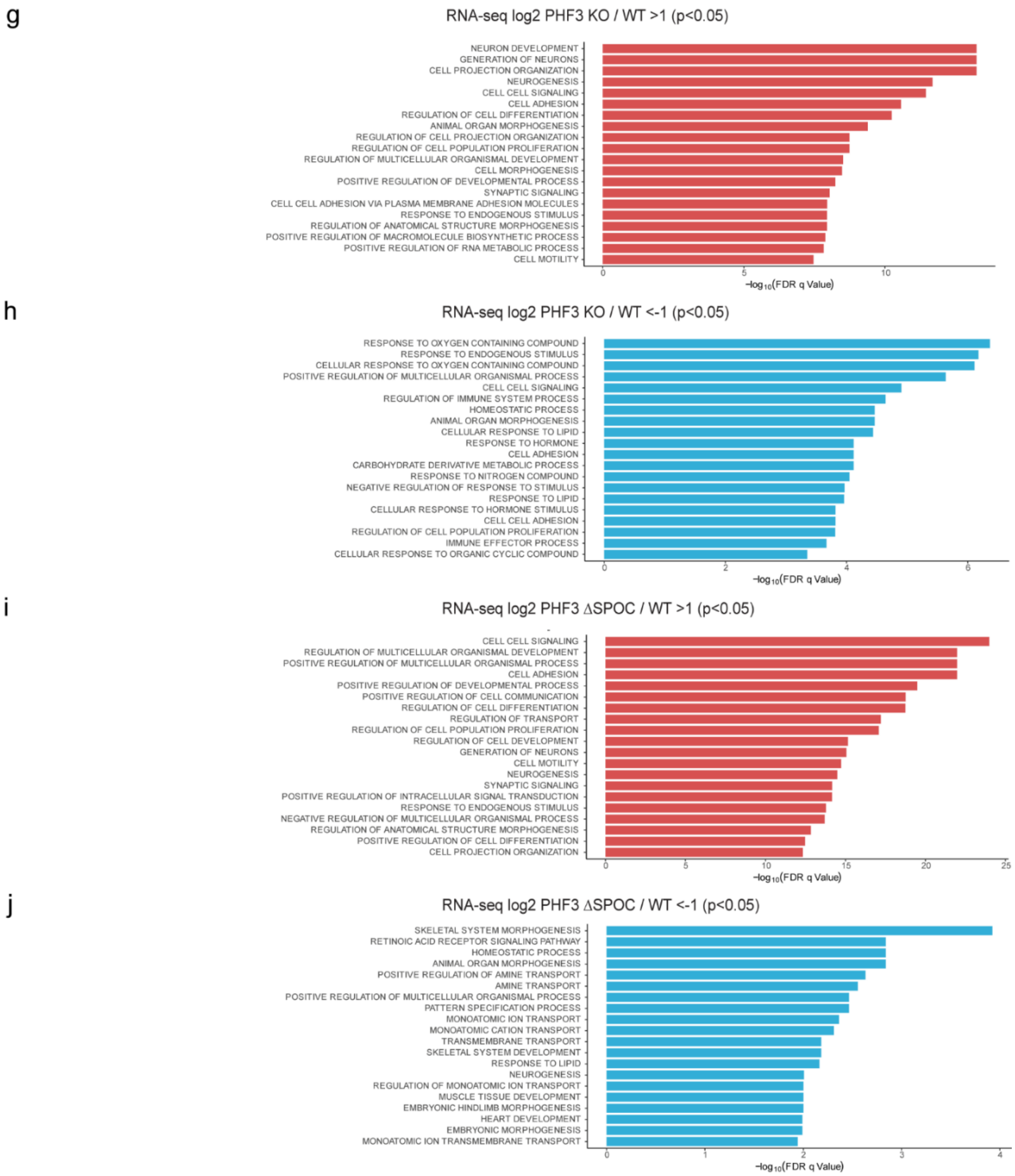

Supplementary Fig. 21: GO analysis of RNA-seq deregulated genes in PHF3 and DIDO HEK293T cells. a Upregulated genes in DIDO Long Isoform KO, **b** Downregulated genes in DIDO Long Isoform KO, **c** Upregulated genes in DIDO full KO, **d** Downregulated genes in DIDO full KO, **e** upregulated genes in DIDO ΔSPOC, **f** downregulated genes in DIDO ΔSPOC, **g** upregulated genes in PHF3 KO, **h** downregulated genes in PHF3 KO, **i** upregulated genes in DIDO ΔSPOC and **j** downregulated genes in DIDO ΔSPOC. GSEA Biological processes tool was used[5].

a

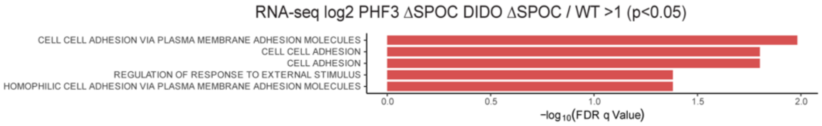
RNA-seq log2 PHF3 ΔSPOC DIDO ΔSPOC / WT >1 (p<0.05)
CELL CELL ADHESION VIA PLASMA MEMBRANE ADHESION MOLECULES
CELL CELL ADHESION
CELL ADHESION
REGULATION OF RESPONSE TO EXTERNAL STIMULUS
HOMOPHILIC CELL ADHESION VIA PLASMA MEMBRANE ADHESION MOLECULES
0.0
0.5
1.0
1.5
2.0
−log10(FDR q Value)

b

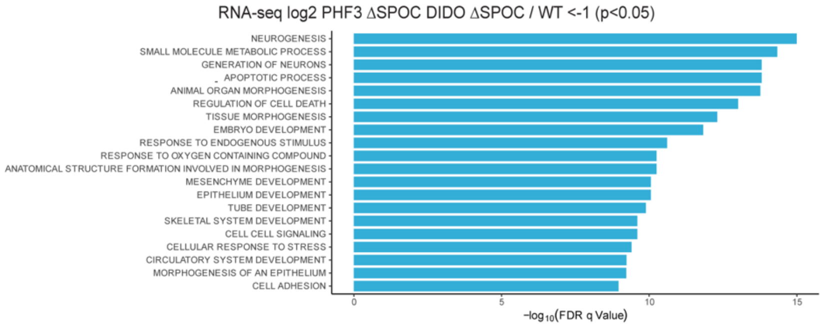
RNA-seq log2 PHF3 ΔSPOC DIDO ΔSPOC / WT <-1 (p<0.05)
NEUROGENESIS
SMALL MOLECULE METABOLIC PROCESS
GENERATION OF NEURONS
APOPTOTIC PROCESS
ANIMAL ORGAN MORPHOGENESIS
REGULATION OF CELL DEATH
TISSUE MORPHOGENESIS
EMBRYO DEVELOPMENT
RESPONSE TO ENDOGENOUS STIMULUS
RESPONSE TO OXYGEN CONTAINING COMPOUND
ANATOMICAL STRUCTURE FORMATION INVOLVED IN MORPHOGENESIS
MESENCHYME DEVELOPMENT
EPITHELIUM DEVELOPMENT
TUBE DEVELOPMENT
SKELETAL SYSTEM DEVELOPMENT
CELL CELL SIGNALING
CELLULAR RESPONSE TO STRESS
CIRCULATORY SYSTEM DEVELOPMENT
MORPHOGENESIS OF AN EPITHELIUM
CELL ADHESION
0
5
10
15
−log10(FDR q Value)

c

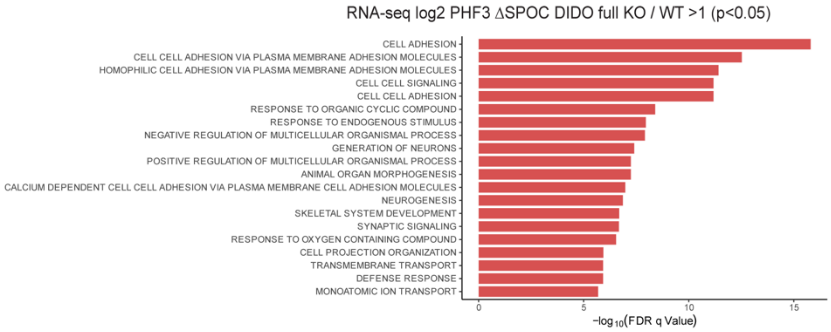
RNA-seq log2 PHF3 ΔSPOC DIDO full KO / WT >1 (p<0.05)
CELL ADHESION
CELL CELL ADHESION VIA PLASMA MEMBRANE ADHESION MOLECULES
HOMOPHILIC CELL ADHESION VIA PLASMA MEMBRANE ADHESION MOLECULES
CELL CELL SIGNALING
CELL CELL ADHESION
RESPONSE TO ORGANIC CYCLIC COMPOUND
RESPONSE TO ENDOGENOUS STIMULUS
NEGATIVE REGULATION OF MULTICELLULAR ORGANISMAL PROCESS
GENERATION OF NEURONS
POSITIVE REGULATION OF MULTICELLULAR ORGANISMAL PROCESS
ANIMAL ORGAN MORPHOGENESIS
CALCIUM DEPENDENT CELL CELL ADHESION VIA PLASMA MEMBRANE CELL ADHESION MOLECULES
NEUROGENESIS
SKELETAL SYSTEM DEVELOPMENT
SYNAPTIC SIGNALING
RESPONSE TO OXYGEN CONTAINING COMPOUND
CELL PROJECTION ORGANIZATION
TRANSMEMBRANE TRANSPORT
DEFENSE RESPONSE
MONOATOMIC ION TRANSPORT
0
5
10
15
−log10(FDR q Value)

d

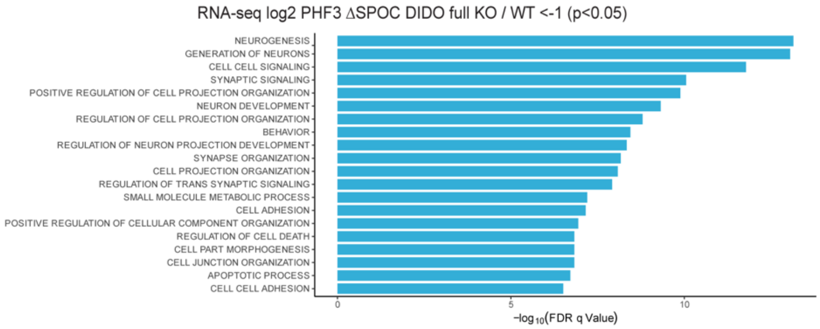
RNA-seq log2 PHF3 ΔSPOC DIDO full KO / WT <-1 (p<0.05)
NEUROGENESIS
GENERATION OF NEURONS
CELL CELL SIGNALING
SYNAPTIC SIGNALING
POSITIVE REGULATION OF CELL PROJECTION ORGANIZATION
NEURON DEVELOPMENT
REGULATION OF CELL PROJECTION ORGANIZATION
BEHAVIOR
REGULATION OF NEURON PROJECTION DEVELOPMENT
SYNAPSE ORGANIZATION
CELL PROJECTION ORGANIZATION
REGULATION OF TRANS SYNAPTIC SIGNALING
SMALL MOLECULE METABOLIC PROCESS
CELL ADHESION
POSITIVE REGULATION OF CELLULAR COMPONENT ORGANIZATION
REGULATION OF CELL DEATH
CELL PART MORPHOGENESIS
CELL JUNCTION ORGANIZATION
APOPTOTIC PROCESS
CELL CELL ADHESION
0
5
10
−log10(FDR q Value)

e

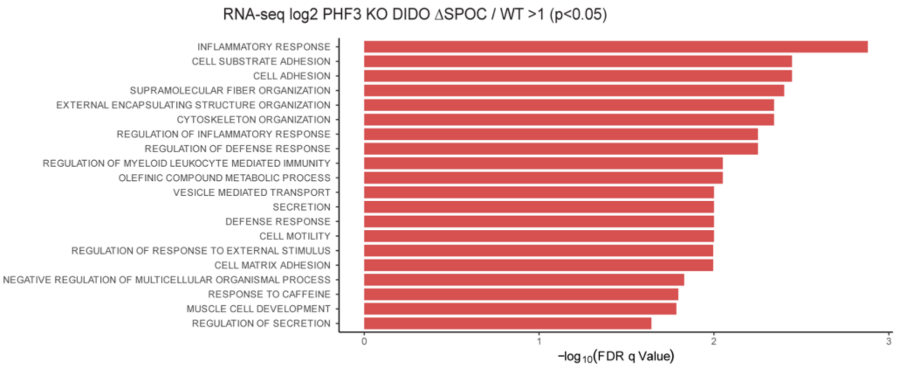

f

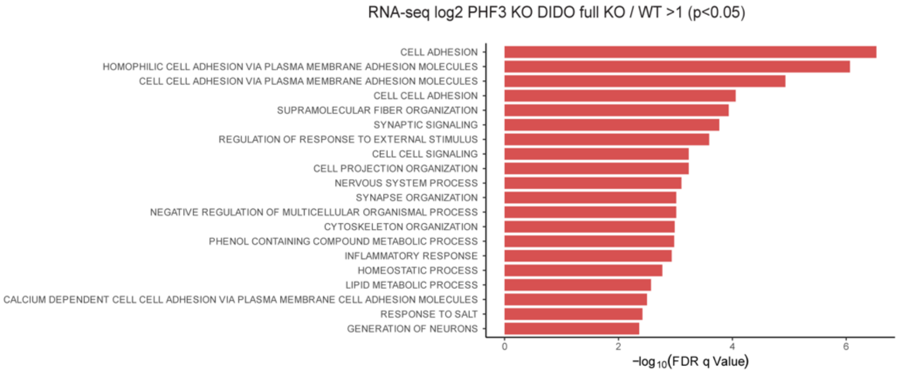

g

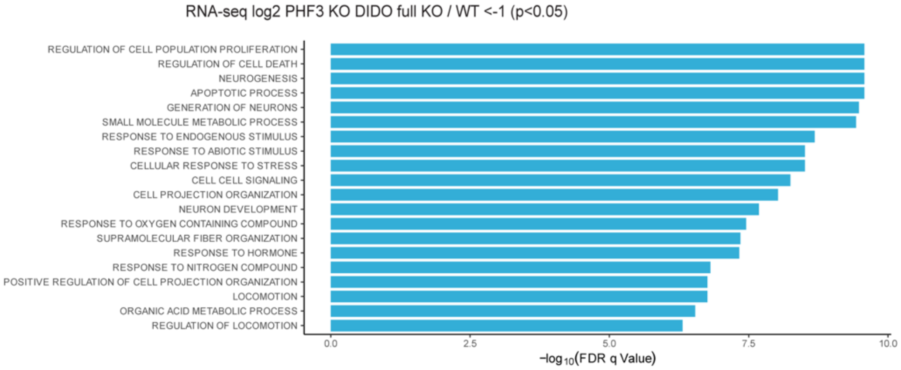

Supplementary Fig. 22: GO analysis of RNA-seq deregulated genes in PHF3 and DIDO double mutant HEK293T cells. **a** Upregulated genes in PHF3 ΔSPOC DIDO ΔSPOC, **b** Downregulated genes in PHF3 ΔSPOC DIDO ΔSPOC, **c** Upregulated genes in PHF3 ΔSPOC DIDO full KO, **d** Downregulated genes in PHF3 ΔSPOC DIDO full KO, **e** Upregulated genes in PHF3 KO DIDO ΔSPOC, **f** Upregulated genes in PHF3 KO DIDO full KO, **g** Downregulated genes in PHF3 KO DIDO full KO. GSEA Biological processes tool was used[5].

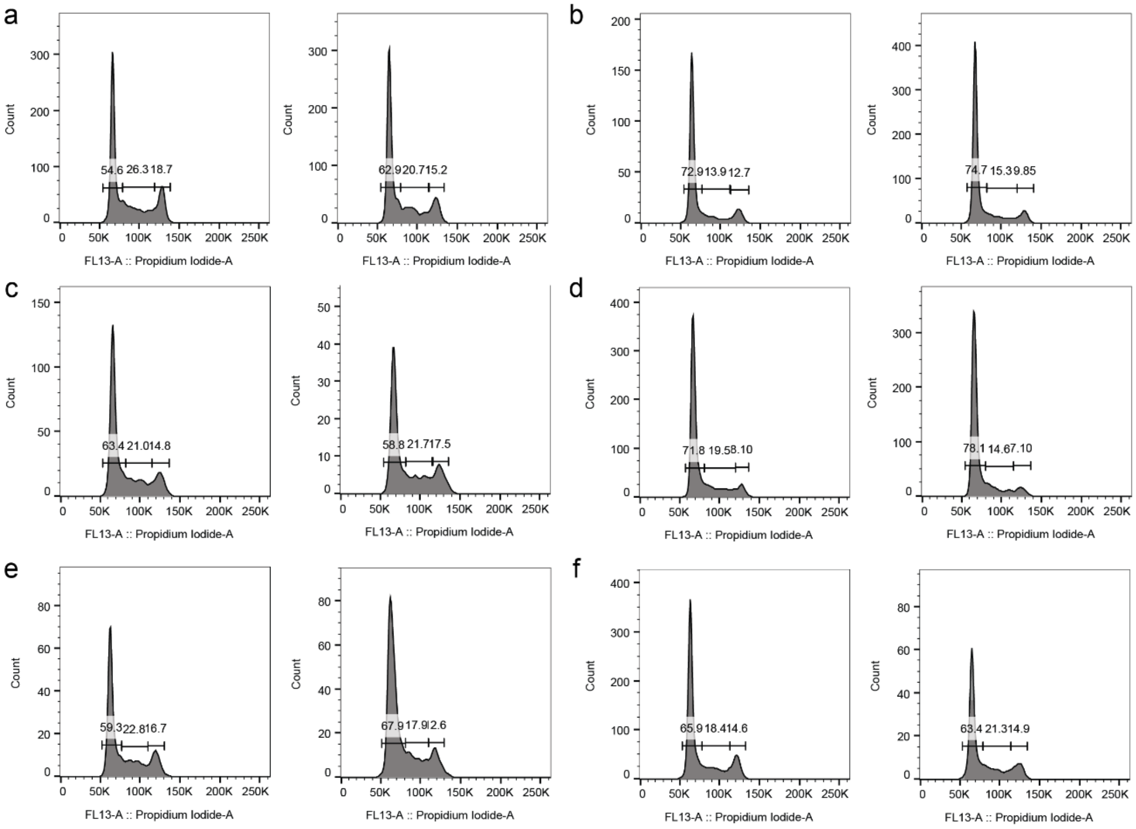

Supplementary Fig. 23: Cell cycle analysis in HEK293T DIDO mutants. Representative histograms from FACS analyses of propidium iodide-stained HEK293T **a** WT, **b** DIDO-[1-88]-Isoform KO, **c** DIDO Long Isoform KO, **d** DIDO full KO, **e** DIDO ΔSPOC and **f** DIDO ΔIDR. Two technical replicates per cell line are shown.

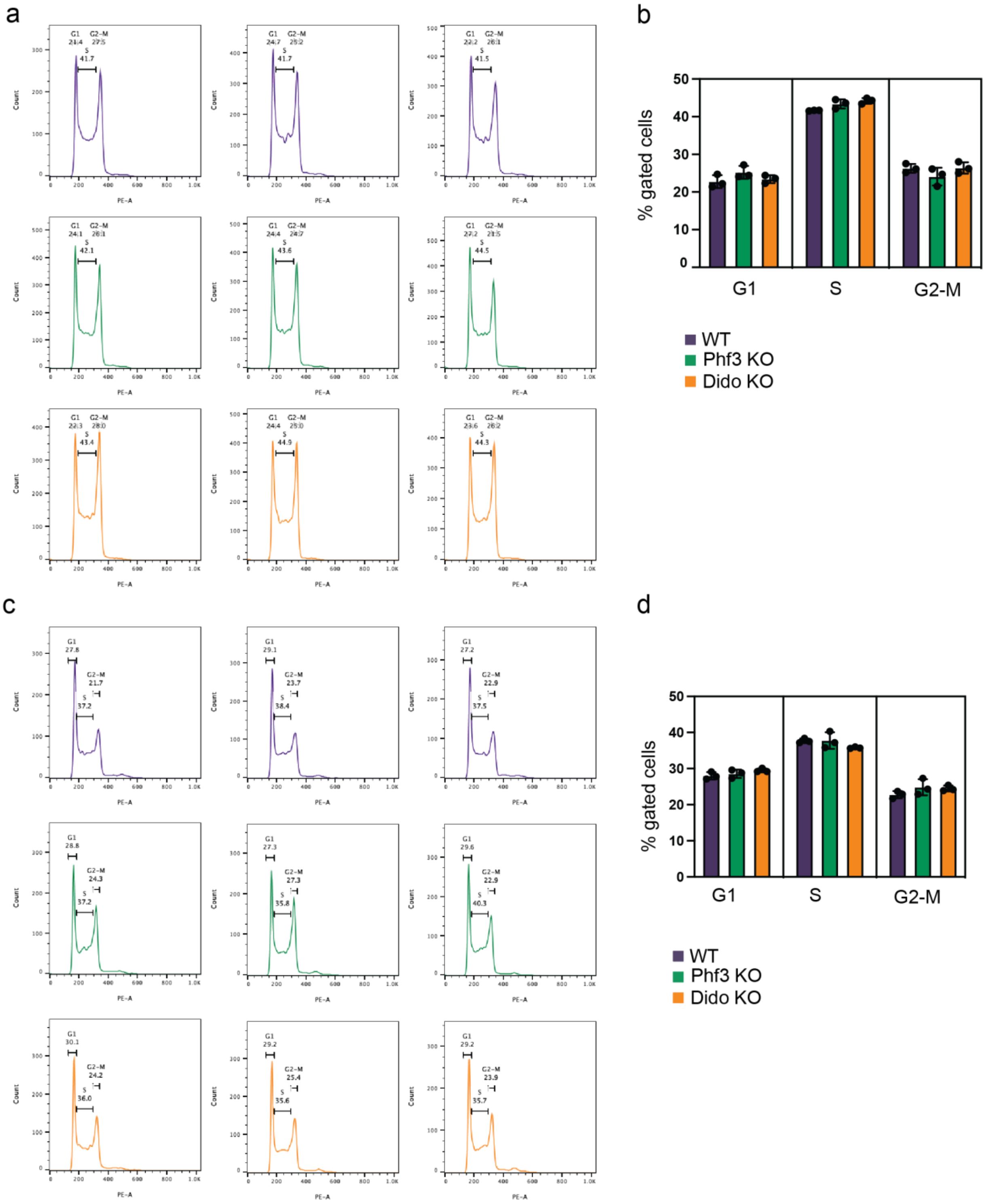

Supplementary Fig. 24: Phf3 and Dido KO mESCs do not display cell cycle differences compared to WT. a,c Histograms from FACS analyses of propidium iodide-stained mESCs. Cells were maintained in **a** DMEM + FBS medium or **b** N2B27 medium. **b,d** Quantification of G1, S and G2 populations from the histograms in **a** and **c** respectively. The experiment was performed in three independent biological replicates. Source data are provided as a Source Data file.

Supplementary References

1 Appel, L. M. *et al.* PHF3 regulates neuronal gene expression through the Pol II CTD reader domain SPOC. *Nature communications* **12**, 6078, doi:10.1038/s41467-021-26360-2 (2021).

2 Appel, L. M. *et al.* The SPOC domain is a phosphoserine binding module that bridges transcription machinery with co- and post-transcriptional regulators. *Nature communications* **14**, 166, doi:10.1038/s41467-023-35853-1 (2023).

3 Lackner, A. *et al.* Cooperative genetic networks drive embryonic stem cell transition from naïve to formative pluripotency. *The EMBO journal* **40**, e105776, doi:10.15252/embj.2020105776 (2021).

4 Ritchie, M. E. *et al.* limma powers differential expression analyses for RNA-sequencing and microarray studies. *Nucleic acids research* **43**, e47, doi:10.1093/nar/gkv007 (2015).

5 Subramanian, A. *et al.* Gene set enrichment analysis: a knowledge-based approach for interpreting genome-wide expression profiles. *Proceedings of the National Academy of Sciences of the United States of America* **102**, 15545-15550, doi:10.1073/pnas.0506580102 (2005).

6 Love, M. I., Huber, W. & Anders, S. Moderated estimation of fold change and dispersion for RNA-seq data with DESeq2. *Genome biology* **15**, 550, doi:10.1186/s13059-014-0550-8 (2014).
