# Supplementary material for: The SPOC proteins DIDO3 and PHF3 co-regulate gene expression and neuronal differentiation
Source: Nat Commun. 2023 Nov 30;14:7912. doi: 10.1038/s41467-023-43724-y (PMC10689479; doi:10.1038/s41467-023-43724-y)
Supplement: Supplementary file 3 — Description of Additional Supplementary Files [file 41467_2023_43724_MOESM3_ESM.pdf]

Description of Additional Supplementary Files

File Name: Supplementary Data 1
Description: Mass spectrometry analysis of the interactome of DIDO isoforms in HEK293T cells.

File Name: Supplementary Data 2
Description: Mass spectrometry analysis of the interactome of Pol II (pS5) in WT and PHF3 mutant HEK293T cells.

File Name: Supplementary Data 3
Description: Mass spectrometry analysis of the interactome of Pol II (pS5) in WT and DIDO mutant HEK293T cells.

File Name: Supplementary Data 4
Description: RNA-seq analysis in WT, PHF3 mutant, DIDO mutant and double mutant HEK293T cells.

File Name: Supplementary Data 5
Description: List of oligonucleotides used in the study.
