# Supplementary material for: The SPOC proteins DIDO3 and PHF3 co-regulate gene expression and neuronal differentiation
Source: Nat Commun. 2023 Nov 30;14:7912. doi: 10.1038/s41467-023-43724-y (PMC10689479; doi:10.1038/s41467-023-43724-y)
Supplement: Supplementary file 9 — Reporting Summary [file 41467_2023_43724_MOESM9_ESM.pdf]

nature portfolio

Corresponding author(s): Dea Slade

Last updated by author(s): Oct 25, 2023

Reporting Summary

Nature Portfolio wishes to improve the reproducibility of the work that we publish. This form provides structure for consistency and transparency in reporting. For further information on Nature Portfolio policies, see our Editorial Policies and the Editorial Policy Checklist.

Statistics

For all statistical analyses, confirm that the following items are present in the figure legend, table legend, main text, or Methods section.

n/a	Confirmed	
☐	☒	The exact sample size (*n*) for each experimental group/condition, given as a discrete number and unit of measurement
☐	☒	A statement on whether measurements were taken from distinct samples or whether the same sample was measured repeatedly
☐	☒	The statistical test(s) used AND whether they are one- or two-sided<br>*Only common tests should be described solely by name; describe more complex techniques in the Methods section.*
☒	☐	A description of all covariates tested
☒	☐	A description of any assumptions or corrections, such as tests of normality and adjustment for multiple comparisons
☐	☒	A full description of the statistical parameters including central tendency (e.g. means) or other basic estimates (e.g. regression coefficient) AND variation (e.g. standard deviation) or associated estimates of uncertainty (e.g. confidence intervals)
☐	☒	For null hypothesis testing, the test statistic (e.g. *F*, *t*, *r*) with confidence intervals, effect sizes, degrees of freedom and *P* value noted<br>*Give P values as exact values whenever suitable.*
☒	☐	For Bayesian analysis, information on the choice of priors and Markov chain Monte Carlo settings
☒	☐	For hierarchical and complex designs, identification of the appropriate level for tests and full reporting of outcomes
☒	☐	Estimates of effect sizes (e.g. Cohen's *d*, Pearson's *r*), indicating how they were calculated

Our web collection on statistics for biologists contains articles on many of the points above.

Software and code

Policy information about availability of computer code

Data collection	RT qPCR was performed on a BioRad CFX Touch cycler operated by BioRad CFX Maestro software (version 2.2).<br>Immunofluorescence images were acquired using an inverse point scanning confocal Zeiss LSM980 Microscope equipped with a Zeiss Plan-Apochromat 63x/1.4 Oil DIC M27 (WD 0.19mm) used for confocal images and Plan-Apochromat 40x/1.4 Oil DIC M27 (WD 0.13mm) objective used for all other images, running with Zeiss ZEN blue 3.3 software (version 3.3.89.0008).<br>For proliferation assays cells were counted on a Countess II Automated Cell Counter (Invitrogen, software version 1.0.249).<br>FACS during cell line generation was performed on a BD FACSMelody cell sorter operated by FACSChorus software (version 1.1.20.0).<br>Cell cycle analysis by FACS was performed on a Bio-Rad ZE5 cell analyzer with excitation laser at 561 nm (50 mW, PI excitation) and a flow rate of 0.1 µL/sec operated by Bio-Rad ZE5 Everest software (version 2.5.0.10).<br>Imaging for LLPS assays was performed on Zeiss Axio Observer Z1 with a 60x oil immersion objective using Zen Blue software (version 3.3.89.0008).
Data analysis	RT qPCR data were analysed and plotted using GraphPad Prism (9.1.1).<br>Chemiluminescent signal from Western Blots was detected on ChemiDoc MP Imaging system (Bio-Rad) operated by Bio-Rad Image Lab Touch Software (version 2.3.0.07). Western Blots were further analysed using Bio-Rad Image Lab Software (version 5.2.1).<br>All immunofluorescence images were processed with Fiji/ImageJ (ImageJ 1.53c) software in parallel with the same Costes-related automatic thresholds for each channel in each individual experiment. Representative 20x20 µm field of views together with regions of interest within nuclei of 2x2 µm were chosen and assembled in OMERO.figure (version 4.3.2) and Adobe Illustrator (version 24.3). Airyscan images were processed for super-resolution with Airyscan filter 6. Colocalization was analysed with the Zeiss co-localization plugin of Zen 3.3 (version 3.3.89.0008). Results were plotted in box and whiskers plots and analysed with two-tailed unpaired Student's t-test with Welch's correction or with one-way ANOVA with Brown-Forsythe & Welch's correction in Prism 9.2.0.<br>RNA-seq and 3'end mRNA-seq data were processed using PiGx-RNA-seq pipeline (version 0.1.0, DOI: 10.1093/gigascience/giy123). Data were

quantified using the GRCh38/hg38 version of the human transcriptome and the dm6 and mm9 versions of the Drosophila (RNA-seq) and mouse (3'end mRNA-seq) spike-in transcriptome (downloaded from the ENSEMBL database, doi:10.1093/nar/gkx1098) using SALMON (version 1.9.0, DOI: 10.1038/nmeth.4197) with default parameters. For visualization purposes, the data was mapped to the GRCh38/hg38, dm6 and mm9 versions of the human, drosophila and mouse genomes using STAR (version 2.7.10a), with the following parameters: --limitOutSJcollapsed 20000000 --limitIObufferSize=1500000000 --outFilterMultimapNmax 10 --seedPerWindowNmax 5. The quantified data was processed using tximport (DOI: 10.12688/f1000research.7563.2), and the differential expression analysis was done using DESeq2 (version 1.38.1, DOI: 10.1186/s13059-014-0550-8).
Proliferation assay data was analysed and plotted using GraphPad Prism (9.1.1).
FACS cell cycle analysis data were analysed in FlowJo (version 10.8.1).
Raw mass spectrometry data from pS5 IP PHF3 samples and FLAG IP with DIDO samples were processed using the MaxQuant software package (version 1.6.0.16) and the Uniprot human reference proteome (July 2018, www.uniprot.org) as well as a database of most common contaminants. Downstream data analysis was performed using the LFQ values in Perseus (version 1.6.2.3). To determine differentially enriched proteins we used the LIMMA package in R (version 3.5.1.) and applied the Benjamini-Hochberg correction for multiple testing to generate adjusted p-values.
For pS5 IP DIDO samples, MS raw data split for each CV using FreeStyle 1.7 (Thermo Fisher) were analysed using the MaxQuant software package (version 2.1.0.0) with the Uniprot human reference proteome (version 2022.01, www.uniprot.org), as well as a database of most common contaminants. MaxQuant output tables were further processed in R 4.2.0 (https://www.R-project.org) using Cassiopeia_LFQ (https://github.com/moritzmadern/Cassiopeia_LFQ). To determine differentially enriched proteins we used the LIMMA package in R (version 3.5.1.) and applied the Benjamini-Hochberg correction for multiple testing to generate adjusted p-values.
Condensate size in LLPS assays was analysed using Fiji (ImageJ 1.53c).

For manuscripts utilizing custom algorithms or software that are central to the research but not yet described in published literature, software must be made available to editors and reviewers. We strongly encourage code deposition in a community repository (e.g. GitHub). See the Nature Portfolio guidelines for submitting code & software for further information.

Data

Policy information about availability of data

All manuscripts must include a data availability statement. This statement should provide the following information, where applicable:

- Accession codes, unique identifiers, or web links for publicly available datasets
- A description of any restrictions on data availability
- For clinical datasets or third party data, please ensure that the statement adheres to our policy

The source data are provided with this paper. 3’end mRNA-sequencing data generated in this study have been deposited in ArrayExpress under accession code: E-MTAB-12757 [https://www.ebi.ac.uk/biostudies/arrayexpress/studies/E-MTAB-12757]. RNA-sequencing data generated in this study have been deposited in ArrayExpress under accession code: E-MTAB-12782 [https://www.ebi.ac.uk/biostudies/arrayexpress/studies/E-MTAB-12782]. The processed RNA-seq data are provided in Supplementary Data 4. Mass spectrometry data have been deposited to the ProteomeXchange Consortium via the PRIDE partner repository47 with the dataset identifier PXD039540 [http://www.ebi.ac.uk/pride/archive/projects/PXD039540] for FLAG IP, PXD039537 [http://www.ebi.ac.uk/pride/archive/projects/PXD039537] for Pol II pS5 IP in PHF3 mutant cells and PXD039567 [http://www.ebi.ac.uk/pride/archive/projects/PXD039567] for Pol II pS5 IP in DIDO mutant cells. The processed mass spectrometry data are provided in Supplementary Data 1-3. Oligonucleotides used in the study are provided in Supplementary Data 5. Genomic DNA sequences were retrieved from Ensembl [https://www.ensembl.org]. Protein sequences were retrieved from UniProt [https://www.uniprot.org].

Research involving human participants, their data, or biological material

Policy information about studies with human participants or human data. See also policy information about sex, gender (identity/presentation), and sexual orientation and race, ethnicity and racism.

Reporting on sex and gender	Not applicable because no human research participants were involved in this study.
Reporting on race, ethnicity, or other socially relevant groupings	Not applicable because no human research participants were involved in this study.
Population characteristics	Not applicable because no human research participants were involved in this study.
Recruitment	Not applicable because no human research participants were involved in this study.
Ethics oversight	Not applicable because no human research participants were involved in this study.

Note that full information on the approval of the study protocol must also be provided in the manuscript.

Field-specific reporting

Please select the one below that is the best fit for your research. If you are not sure, read the appropriate sections before making your selection.

☒ Life sciences ☐ Behavioural & social sciences ☐ Ecological, evolutionary & environmental sciences

For a reference copy of the document with all sections, see nature.com/documents/nr-reporting-summary-flat.pdf

Life sciences study design

All studies must disclose on these points even when the disclosure is negative.

Sample size	No statistical methods were used to predetermine sample size. Sample sizes were chosen based on our previous experience and previously published similar studies (e.g. https://doi.org/10.1038/s41467-021-26360-2, https://doi.org/10.1038/s41467-023-35853-1).<br>RT qPCR was performed in 3-5 biological replicates and each individual sample was measured in technical triplicates. This sample size was chosen because it allows for statistical analysis and detection and removal of potential outliers. Since we were in all cases able to replicate our results, we deemed the sample size sufficient.<br>Sample sizes and sequencing depth for NGS experiments were chosen based on the standards recommended by the ENCODE Consortium and on previously published similar studies. ENCODE recommends two or more biological replicates for RNA-seq. We chose to perform RNA-seq and 3'polyA mRNA-seq in 3 biological replicates because this allows for detection and removal of potential outliers. All attempts at replication of NGS data were successful, therefore we consider the sample size sufficient.<br>Proliferation assays, FACS cell cycle analysis, Co-IPs followed by mass spectrometry, Co-IPs followed by Western Blotting and Airyscan colocalization analysis experiments were performed in triplicates (with multiple cells per replicate being analysed for Airyscan colocalization analysis) because this allows for statistical analysis and detection and removal of potential outliers. Since we were in all cases able to replicate our results in all three replicates, we deemed the sample size sufficient.<br>Sucrose gradient ultracentrifugation to show DIDO-PHF3 complex formation was performed once, but complex formation in intact cells was further assessed by microscopy-based colocalization assays.<br>For LLPS assays at least 250 droplets per condition were imaged, the sample size was chosen based on previously published similar studies.<br>Neuronal differentiation experiments were performed in 4 biological replicates to allow for detection and removal of potential outliers. Since the results were replicated in all replicates we consider the sample size sufficient.<br>Genotyping PCRs, Western blots and immunofluorescence during cell line generation were performed once, since they were meant to confirm the genotype, protein expression and protein localization and not to generate experimental data.
Data exclusions	No data were excluded from analysis.
Replication	All attempts at replication were successful. RT qPCR was performed in 3-5 biological replicates with similar results (the precise number of biological replicate for each experiment is provided in the corresponding figure legend), each individual sample was measured in technical triplicates.<br>Mass spectrometry experiments were performed in three biological replicates, results were successfully replicated. Co-IP experiments followed by Western blotting were performed to confirm results from mass spectrometry analysis. These experiments were performed once, they confirmed the interactors identified by mass spectrometry.<br>Sucrose gradient ultracentrifugation to show DIDO-PHF3 complex formation was performed once. We did not replicate this experiment because we also assessed complex formation in intact cells using a different, independent method through microscopy-based colocalization assays. Colocalization assays were performed in biological triplicates with multiple cells imaged per replicate. Both methods independently confirmed complex formation.<br>LLPS assays were performed in two independent replicates with at least 250 droplets imaged per replicate (precise number of droplets imaged is provided in the figure legend). The results were successfully replicated.<br>RNA-seq experiments were performed in three biological replicates and were successfully replicated.<br>Immunofluorescence analysis was performed in two biological replicates with multiple cells imaged per replicate. The results were successfully replicated.<br>Proliferation assays were performed in three biological replicates with similar results.<br>FACS cell cycle analysis was performed in three biological replicates with similar results.<br>Neuronal differentiation assays were performed in four biological replicates with similar results.<br>Experiments meant to confirm successful CRISPR/Cas9 editing rather than generate experimental data (genotyping PCRs, Western Blots, IF) were not replicated.
Randomization	Randomization was not performed for this study. Samples (cell lines) were allocated to experimental groups based on genotype and paired with a control, i.e. KO and domain deletion cell lines were compared to a control WT cell line or cells transfected with FLAG- or HA-constructs were compared to empty vector transfected control cells. All cell lines were grown under the same conditions and experiments were performed under identical conditions.
Blinding	The investigators were not blinded in this study since they were involved in the planning, execution and analysis of the experiments. Within-experiment sample groups and the respective controls were prepared, processed and analysed at the same time and under identical conditions to eliminate any bias, so prior knowledge had no impact on data output. No subjective process was involved in the analysis of the data. Analysis of NGS- and mass spectrometry data was carried out bioinformatically applying the same parameters to all samples without need for investigator blinding.

Reporting for specific materials, systems and methods

We require information from authors about some types of materials, experimental systems and methods used in many studies. Here, indicate whether each material, system or method listed is relevant to your study. If you are not sure if a list item applies to your research, read the appropriate section before selecting a response.

Materials & experimental systems

n/a	Involved in the study
☐	☒ Antibodies
☐	☒ Eukaryotic cell lines
☒	☐ Palaeontology and archaeology
☒	☐ Animals and other organisms
☒	☐ Clinical data
☒	☐ Dual use research of concern
☒	☐ Plants

Methods

n/a	Involved in the study
☒	☐ ChIP-seq
☐	☒ Flow cytometry
☒	☐ MRI-based neuroimaging

Antibodies

Antibodies used

The antibody dilutions used are provided in Supplementary Table 1.
Rabbit anti-CK2α (polyclonal), Cell Signaling 2656
Rabbit anti-DEK mAb clone E4S5J, Cell Signaling 29812
Rabbit anti-DIDO1 (polyclonal), Atlas Antibody HPA0449904
Rabbit anti-Pan DIDO (polyclonal), Millipore ABN1367
Mouse anti-Human DIDO1 cl. 734823, R&D Systems MAB6947
Mouse anti-FLAG cl. M2, Sigma F1804
Mouse anti-FLAG M2-peroxidase, Sigma A8592
Rabbit anti-GFP (polyclonal), abcam ab290
Mouse anti-GFP cl. 13.1 and cl. 7.1 (mixture of two monoclonal antibodies), Roche 11814460001
Rabbit anti-H3K9ac (polyclonal), Sigma 07-352
Rabbit anti-H3K9me3 (polyclonal), abcam ab8898
Rabbit anti-fibrillarin cl. C13C3, Cell Signaling 2639
Mouse anti-HA.11 cl. 16B12, Covance 901513
Rabbit anti-HCFC1 (polyclonal), Cell Signaling 69690
Rabbit anti-histone macro H2A.1 (polyclonal), abcam ab37264
Rabbit anti-histone macro H2A1.2 (polyclonal), Cell Signaling 4827
Rabbit anti-H2AZ (polyclonal), Cell Signaling 2718
Rabbit anti-gH2AX (polyclonal), Bethyl A300-081A
Rabbit anti-PAF1 (polyclonal), abcam ab20662
Mouse anti-PARP cl. C2-10, Trevigen 4338-MC-50
Mouse anti-PARP2 cl. 4G8, Enzo ALX-804-639
Rabbit anti-PHF3 (polyclonal), Atlas antibodies HPA025763
Rat anti-pS2 Pol II cl. 3E10, Monoclonal antibody facility (Helmholtz Center, Munich)
Rat anti-pS5 Pol II cl. 3E8, Monoclonal antibody facility (Helmholtz Center, Munich)
Rat anti-RNA polymerase II pS7 cl. 4E12, Millipore 04-1570
Mouse anti-pS5 Pol II clone 4H8 Cell Signaling 2629
Mouse anti-Pol II clone F-12 Santa Cruz sc-55492
Mouse anti-SPT5 clone 17 BD 611107
Rabbit anti-SPT6 (polyclonal), Novus Biologicals NB100-2582
Mouse anti-α-tubulin cl. B-5-1-2, Sigma T6074
Rabbit anti-β-Tubulin III TuJ1 (polyclonal), Sigma T2200
Mouse anti-GFAP cl. G-A-5, Sigma G3893
Rabbit anti-YB1 (polyclonal), Cell Signaling D299
Rat anti-ZNF768 cl. 5c8, AG Eick (source: doi: 10.1093/nar/gky1148)
Rabbit anti-TBP (D5C9H) XP, Cell Signaling 44059
Goat anti-rabbit AF488 (polyclonal), ThermoFisher A11008
Goat anti-mouse AF488 (polyclonal), ThermoFisher A11001
Goat anti-rabbit AF568 (polyclonal), ThermoFisher A11011
Goat anti-mouse AF568 (polyclonal), ThermoFisher A11004
Goat anti-rat AF594 (polyclonal), ThermoFisher A11007
Goat anti-rat AF647 (polyclonal), abcam ab150167
Goat anti-rabbit AF647 (polyclonal), ThermoFisher A21244

Validation

Rabbit anti-CK2α (Cell Signaling 2656): Used in >30 publications. Statement from the manufacturer: This antibody detects endogenous levels of total CK2α 1 protein. This antibody may cross-react with CK2α prime. An image of Western Blot detection of CK2α in lysates from A673, HT1376, HeLa and NIH/3T3 cell lines is provided on the manufacturer's website. This antibody is further validated for Western Blotting in human cells in Hussein et al. 2021 (https://doi.org/10.3390/cells10071770) where it is used to confirm knock-down of CK2α in U2OS and KHOS/NP cells.
Rabbit anti-DEK cl. E4S5J (Cell Signaling 29812). Statement from the manufacturer: DEK (E4S5J) Rabbit mAb recognizes endogenous levels of total DEK protein. Non-specific staining has been observed by IHC in nerve cells. Western Blot images are provided by the manufacturer, the antibody detects DEK in wild type 293T, but not DEK KO 293T cells.
Rabbit anti-DIDO1 (Atlas antibodies HPA049904): Manufacturer's statement on validation: Validated in Western blot using relevant lysates. An image of Western Blot detection in the human cell line RT-4 using this antibody is shown on the manufacturer's website. Target specificity was validated in this study and our previous study (https://doi.org/10.1038/s41467-023-35853-1), where it was used to detect loss of DIDO protein after CRISPR/Cas9 knock-out by Western Blotting and IF.
Rabbit anti-Pan DIDO (Millipore ABN1367): Manufacturer's statement on specificity: This polyclonal antibody detects an N-terminal epitope present in all four spliced isoforms of human DIDO1 reported by UniProt (Q9BTC0). Image showing Western Blot detection of

DIDO isoforms in HepG2 cell lysate is provided on the manufacturer's website.
Mouse anti-Human DIDO1 (R&D Systems MAB6947): Manufacturer's statement on specificity testing by Western Blot: Detection of Human DIDO1 by Western Blot. Western blot shows lysates of Raji human Burkitt's lymphoma cell line. Gels were loaded with 30 µg of whole cell lysate (WCL), 20 µg of cytoplasmic (Cyto), and 10 µg of nuclear (Nuc) extracts. PVDF membrane was probed with 2 µg/mL of Mouse Anti-Human DIDO1 Monoclonal Antibody (Catalog # MAB6947) followed by HRP-conjugated Anti-Mouse IgG Secondary Antibody (Catalog # HAF007). A specific band was detected for DIDO1 at approximately 280 kDa (as indicated).
Mouse anti-FLAG cl. M2 (Sigma F1804): Statement from the manufacturer: For highly sensitive and specific detection of FLAG fusion proteins by immunoblotting, immunoprecipitation (IP), immunohistochemisty, immunofluorescence and immunocyotchemistry. Optimized for single banded detection of FLAG fusion proteins in mammalian, plant, and bacterial expression systems. Image of immunofluorescence detection of a FLAG-tagged protein is provided on the manufacturer's website. Validated for IF in human cells in DOI 10.1186/s12985-016-0610-7, where FLAG-tagged proteins expressed in HeLa cells were detected by IF using mock transfected cells as a negative control.
Mouse anti-FLAG M2-peroxidase (Sigma A8592): used for Western Blotting in >50 publications (e.g. e.g. https://doi.org/10.1038/s41467-021-26360-2, https://doi.org/10.1038/s41467-023-35853-1) and validated in this study (Fig. 6c and 7d, empty vector vs. FLAG-tagged constructs).
Rabbit anti-GFP (Abcam ab290): Manufacturer's statement: Anti-GFP antibody (ab290) is a highly versatile antibody that gives a stronger signal than other anti-GFP antibodies available. On Western blot the antibody detects the GFP fraction from cell extracts expressing recombinant GFP fusion proteins and has also been shown to be useful on mouse sections fixed with formalin. In Immunocytochemistry, the antibody gives a very good signal on recombinant YES-GFP chimeras expressed in COS cells (McCabe et al. 1999 and figure below). It is routinely used in Immunoprecipitation (IP) and IP-Western protocols and has been used successfully in HRP Immunohistochemistry at 1:200 on whole-mount mouse embryos.
Mouse anti-GFP (Roche 11814460001): Manufacturer's statement on quality control: Anti-GFP is tested for functionality and purity relative to a reference standard to confirm the quality of each new reagent preparation. Validated for Immunofluorescence in DOI:10.1093/cvr/cvs277 and https://doi.org/10.1242/dev.043703.
Rabbit anti-H3K9ac (Sigma 07-352): Manufacturer's statement: Broad species-cross reactivity is expected. Recognizes acetyl-histone H3 (Lys9), Mr 17 kDa. An additional unknown protein was detected at Mr 23 kDa. Images demonstrating detection of H3K9ac by immunofluorescence are provided on the manufacturer's website. Detection of H3K9ac in human cells was demonstrated e.g. in doi: 10.1111/cas.12717.
Rabbit anti-H3K9me3 (abcam ab8898): Manufacturer's statement: Histone H3 (tri methyl K9) antibody (ab8898) is specific for Histone H3 tri methyl Lysine 9. Shows slight cross-reactivity with tri methyl K27, which shares a similar epitope. Does not react with mono or di methylated K9. Validated for Immunofluorescence by the manufacturer in Mouse 3T3MEF, Indian muntjac fibroblast cells, HeLa cells and Mouse Embryonic Stem cells. Immunofluorescence images for HeLa cells are provided on the manufacturer's website.
Rabbit anti-fibrillarin (C13C3) (Cell Signaling 2639): Manufacturer's statement: Fibrillarin (C13C3) Rabbit mAb detects endogenous levels of total fibrillarin protein. Immunofluorescence images for mouse tissues and HeLa cells are provided on the manufacturer's website.
Mouse anti-HA.11 cl. 16B12 (Covance 901513): Manufacturer's statement on quality control: Each lot of this antibody is quality control tested by Western blotting. Validated by the manufacturer for Western Blotting and IF (images of Western Blot and IF detection in cell lines transfected with and HA-construct using non-transfected cells as a control are provided on the manufacturer's website) and in this study (Fig. 7e,f; detection of HA-constructs expressed in HEK293T cells using empty vector transfected cells as a negative control).
Rabbit anti-HCFC1 (Cell Signaling 69690): Manufacturer's statement on specificity: HCFC1 Antibody (Amino-terminal Antigen) recognizes endogenous levels of total HCFC1 protein. This antibody also recognizes amino-terminal fragments (HCFC1-N) resulting from O-GlcNAc transferase (OGT) cleavage. Western Blot image of HCFC1 detection in lysates from human cells is provided on the manufacturer's website.
Rabbit anti-histone macro H2A.1 (abcam ab37264): Manufacturer's statement on specificity: ab37264 recognises the three known isoforms of mH2A1 including mH2A1.2 (longest isoform) and the mH2A1.1 (shortest isoform). Images for Western Blot detection in human cell lines HeLa and HepG2 are provided on the manufacturer's website. Target specificity validated by the manufacturer using HAP1 WT and KO cells, Western blot image provided on the manufacturer's website.
Rabbit anti-histone macro H2A1.2 (Cell Signaling 4827): Manufacturer's statement on specificity: MacroH2A1.2 Antibody detects endogenous levels of the core histone MacroH2A1.2 protein (MacroH2A1, isoform 2). The antibody does not cross-react with MacroH2A1.1 (MacroH2A1, isoform 1), MacroH2A2 or histone H2A. Images showing Western Blot detection in human cell lysates (HeLa, H-4-II-E) and MacroH2A1.2 transfected cells are provided on the manufacturer's website.
Rabbit anti-H2AZ (Cell Signaling, 2718): Manufacturer's statement on specificity: Histone H2A.Z Antibody detects endogenous levels of histone H2A.Z protein. The antibody does not cross-react with other histone proteins, including histone H2A. Western Blot image demonstrating detection of H2A.Z in cell lysate from human cell lines as well as recombinant H2A.Z, but not recombinant H2A, is provided on the manufacturer's website.
Rabbit anti-gH2AX (Bethyl A300-081A): Validate by the manufacturer, images of Western Blot detection before and after treatment with etoposide are provided on the manufacturer's website.
Rabbit anti-PAF1 (Abcam ab20662): Manufacturer's statement (WB): 'ab20662 recognizes a band at approximately 80kDa, which corresponds in size to PAF1 / PD2. Although it has a calculated molecular weight of ~60kDa, several groups have shown that it migrates at a size of 80-90kDa (see Moniaux, et.al., Oncogene Feb. 2006, Yart et.al., Mol Cell Biol 25:5052-60, 2005, and Rozenblatt-Rosen, et.al., Mol Cell Biol 25:612-20, 2005). There is also a smaller band at ~73kDa which may be a degradation product. Both bands are competed away by the addition of immunizing peptide, suggesting that the interaction is specific.'
Mouse anti-PARP cl. C2-10 (Trevigen 4338-MC-50): Manufacturer's statement on specificity: This antibody recognizes the full length (116 kDa) and 85 kDa apoptosis-related and necrosis-related 50kDa, 62 kDa and 74 kDa cleavage fragments of PARP in human, monkey, mouse, rat, bovine and hamster systems. Chicken PARP is not recognized. Other species have not been tested. An image of Western Blot detection of PARP in human cell lines (Jurkat, CCRF-CEM) is provided in the antibody data sheet.
Mouse anti-PARP2 cl. 4G8 (Enzo ALX-804-639): Manufacturer's statement of specificity: Recognizes an epitope in domain E of PARP-2. Image of Western Blot detection in HeLa cells is provided on the manufacturer's website.
Rabbit anti-PHF3 (Atlas antibodies HPA025763): Validated in our previous study (https://doi.org/10.1038/s41467-021-26360-2), where it was used to detect loss of PHF3 protein after CRISPR/Cas9 knock-out by Western Blotting. Statement from the manufacturer: All Prestige Antibodies Powered by Atlas Antibodies are developed and validated by the Human Protein Atlas (HPA) project and as a result, are supported by the most extensive characterization in the industry. The Human Protein Atlas project can be subdivided into three efforts: Human Tissue Atlas, Cancer Atlas, and Human Cell Atlas. The antibodies that have been generated in support of the Tissue and Cancer Atlas projects have been tested by immunohistochemistry against hundreds of normal and disease

tissues and through the recent efforts of the Human Cell Atlas project, many have been characterized by immunofluorescence to map the human proteome not only at the tissue level but now at the subcellular level. These images and the collection of this vast data set can be viewed on the Human Protein Atlas (HPA) site by clicking on the Image Gallery link.
Rat phospho-CTD antibodies cl. 3E8, cl. 3E10 and cl. 4E12 were validated in DOI: 10.1126/science.1145977
Mouse anti-Pol II 4H8 (Cell Signaling) - Manufacturer's statement on Specificity: 'Rpb1 CTD (4H8) Antibody detects endogenous levels of total Rpb1 protein (both phosphorylated and unphosphorylated forms).' Images confirming Western Blot detection in human cells are provided on the manufacturer's website.
Mouse anti-Pol II clone F-12 (Santa Cruz sc-55492): used in >30 publications and validated in our previous study (https://doi.org/10.1038/s41467-021-26360-2) using purified Pol II complex in WB and in ChIP.
Mouse anti-SPT5 (Becton Dickinson 611107) - Manufacturer's statement: 'This antibody is routinely tested by western blot analysis.'
Rabbit anti-SPT6 (Novus Biologicals NB100-2582): Validated by the manufacturer using siRNA knockdown to confirm the specificity of Spt6 antibody in C2C12 myoblasts. The antibody was validated for Western Blotting in human cells in Lu et al. 2020 (https://doi.org/10.1172/JCI138577), where it was used to confirm SPT6 depletion in MDA-MB-231 cells transfected with shRNAs targeting SPT6 by Western Blotting.
Mouse anti-α-tubulin (Sigma T6074): The antibody's target specificity was validated by the manufacturer's 'Antibody Enhanced Validation - Independent Antibody Verification' procedure. Manufacturer's statement on the procedure: Demonstrating antibody specificity through the use of multiple antibodies against target in IHC or ICC. Expected results: All antibodies should show similar staining patterns or experimental results. The validity of results obtained with an antibody in a given immunoapplication may be supported by showing that the same results are obtained using the identical protocol with a different antibody raised against the same target. At least two antibodies with non-overlapping epitopes are applied across a panel of samples, such as sections from the same tissue. This approach has the added advantage of enabling validation of both antibodies used for comparison of binding characteristics. Images displaying Western blot detection of α-tubulin in lysate from human cell lines (HeLa, Jurkat) are shown on the manufacturer's website and the antibody was used in >100 publications.
Rabbit anti-β-Tubulin III TuJ1 (Sigma T2200): Statement by the manufacturer: Anti-β-Tubulin III recognizes human, mouse, and rat β-tubulin III. Images of immunofluorescence detection in mouse cells are provided on the manufacturer’s website. Target specificity was validated by the manufacturer using a KO cell line (Western Blot images provided on the manufacturer’s website).
Mouse anti-GFAP (Sigma G3893): Manufacturer's statement: The antibody reacts specifically with GFAP in immunoblotting assays and labels astrocytes, Bergmann glia cells and chondrocytes of elastic cartilage in immunohistochemical staining. The antibody reacts with glial specific antigen in frozen or alcohol-fixed tissue sections. Images demonstrating immunofluorescence staining are provided on the manufacturer's website.
Rabbit anti-YB1 (Cell Signaling D299): Statement by the manufacturer: YB1 (D299) Antibody detects endogenous levels of total YB1 protein. Western blot images of YB1 detection in human cells (MCF-7) are provided on the manufacturer’s website.
Rat anti-ZNF768: provided by Dirk Eick, validated in doi: 10.1093/nar/gky1148
Rabbit anti-TBP (D5C9H) XP (Cell Signaling 44059): Manufacturer's statement: TBP (D5C9H) XP Rabbit mAb recognizes endogenous levels of total TBP protein. Images of Western Blot detection in human cell lines (HeLa, H-4-II-E) are provided by the manufacturer.
Goat anti-rabbit Alexa Fluor 488 (Thermo Fisher A11008): Manufacturer’s statement on antibody testing in IF in human cells: Immunofluorescence analysis of Goat anti-Rabbit IgG (H+L) Cross-Adsorbed Secondary Antibody Alexa Fluor® 488 conjugate was performed using HeLa cells stained with alpha Tubulin Rabbit Polyclonal Antibody (Product # PA5-16891). The cells were fixed with 4% paraformaldehyde for 10 minutes, permeabilized with 0.1% Triton™ X-100 for 10 minutes, blocked with 1% BSA for 1 hour and labeled with 2 µg/mL Rabbit primary antibody for 3 hours at room temperature. Goat anti-Rabbit IgG (H+L) Cross-Adsorbed Secondary Antibody Alexa Fluor® 488 conjugate (Product # A-11008) was used at a concentration of 4 µg/mL in phosphate buffered saline containing 0.2% BSA for 45 minutes at room temperature, for detection of alpha Tubulin in the cytoplasm (Panel a: green). Nuclei (Panel b: blue) were stained with DAPI in SlowFade® Gold Antifade Mountant (Product # S36938). F-actin was stained with Rhodamine Phalloidin (Product # R415, 1:300) (Panel c: red). Panel d represents the composite image. No nonspecific staining was observed with the secondary antibody alone (panel f), or with an isotype control (panel e). The images were captured at 60X magnification.
Goat anti-mouse Alexa Fluor 488 (Thermo Fisher A11001): Manufacturer’s statement on antibody testing in IF in human cells: Golgi and actin staining in HeLa cells. Golgi in fixed and permeabilized HeLa cells labeled with anti-golgin-97 monoclonal antibody (Product # A-21270) and visualized with green-fluorescent Alexa Fluor® 488 Goat-Anti-Mouse IgG (Product # A-11001). Actin was stained with red-fluorescent Alexa Fluor® 594 phalloidin (Product # A12381); nuclei were stained with blue-fluorescent DAPI (Product # D1306, D3571, D21490). Treatment with Image-iT® FX signal enhancer (Product # I36933) largely eliminates nonspecific dye binding (bottom) as compared to untreated slide (top).
Goat anti-rabbit Alexa Fluor 568 (Thermo Fisher A11011): Manufacturer’s statement on antibody testing in IF in human cells: Immunofluorescence analysis of Goat anti-Rabbit IgG (H+L) Cross-Adsorbed Secondary Antibody Alexa Fluor® 568 conjugate was performed using HeLa cells stained with alpha Tubulin Rabbit Polyclonal Antibody (Product # PA5-16891) The cells were fixed with 4% paraformaldehyde for 10 minutes, permeabilized with 0.1% Triton™ X-100 for 10 minutes, blocked with 1% BSA for 1 hour and labeled with 2 µg/mL primary antibody for 3 hours at room temperature. Goat anti-Rabbit IgG (H+L) Cross-Adsorbed Secondary Antibody Alexa Fluor® 568 conjugate (Product # A-11011) was used at a concentration of 2 µg/mL in phosphate buffered saline containing 0.2% BSA for 45 minutes at room temperature, for detection of alpha Tubulin in the cytoplasm (Panel a: red). Nuclei (Panel b: blue) were stained with DAPI in SlowFade® Gold Antifade Mountant (Product # S36938). F-actin was stained with Alexa Fluor® 488 Phalloidin (Product # A12379), 1:300) (Panel c: green). Panel d represents the composite image. No nonspecific staining was observed with the secondary antibody alone (panel f), or with an isotype control (panel e). The images were captured at 60X magnification.
Goat anti-mouse Alexa Fluor 568 (Thermo Fisher A11004): Manufacturer’s statement on antibody testing in IF in human cells: Immunofluorescence analysis of Goat anti-Mouse IgG (H+L) Cross-Adsorbed Secondary Antibody, Alexa Fluor® 568 conjugate was performed using HeLa cells stained with alpha Tubulin (236-10501) Mouse Monoclonal Antibody (Product # A11126). The cells were fixed with 4% paraformaldehyde for 10 minutes, permeabilized with 0.1% Triton™ X-100 for 10 minutes, blocked with 1% BSA for 1 hour and labeled with 2 µg/mL primary antibody for 3 hours at room temperature. Goat anti-Mouse IgG (H+L) Cross-Adsorbed Secondary Antibody, Alexa Fluor® 568 (Product # A-11004) was used at a concentration of 2 µg/mL in phosphate buffered saline containing 0.2% BSA for 45 minutes at room temperature, for detection of alpha Tubulin in the cytoplasm (Panel a: red). Nuclei (Panel b: blue) were stained with DAPI in SlowFade® Gold Antifade Mountant (Product # S36938). F-actin was stained with Alexa Fluor® 488 Phalloidin (Product # A12379), 1:300) (Panel c: green). Panel d represents the composite image. No nonspecific staining was observed with the secondary antibody alone (panel f), or with an isotype control (panel e). The images were captured at 60X magnification.
Goat anti-rat AF594 (ThermoFisher A11007): Manufacturer’s statement on antibody testing in IF in human cells: Immunofluorescence analysis of Goat anti-Rat IgG (H+L) Secondary Antibody, Alexa Fluor 594 conjugate was performed using A549

cells stained with alpha Tubulin (YL1/2) Rat Monoclonal Antibody (Product # MA1-80017). The cells were fixed with 4% paraformaldehyde for 10 minutes, permeabilized with 0.1% Triton™ X-100 for 10 minutes, blocked with 1% BSA for 1 hour and labeled with 2µg/mL Rat primary antibody for 3 hours at room temperature. Goat anti-Rat IgG (H+L) Secondary Antibody, Alexa Fluor 594 conjugate (Product # A-11007) was used at a concentration of 4µg/mL in phosphate buffered saline containing 0.2 % BSA for 45 minutes at room temperature, for detection of alpha Tubulin in the cytoplasm (Panel a: red). Nuclei (Panel b: blue) were stained with DAPI in SlowFade® Gold Antifade Mountant (Product # S36938). F-actin was stained with Alexa Fluor® 488 Phalloidin (Product # A12379), 1:300) (Panel c: green). Panel d represents the composite image. No nonspecific staining was observed with the secondary antibody alone (panel f), or with an isotype control (panel e). The images were captured at 60X magnification.
Goat anti-rat AF647 (abcam ab150167): Manufacturer's statement: By immunoelectrophoresis and ELISA this antibody reacts specifically with rat IgG and with light chain common to other rat immunogobulins. No antibody was detected against non-immunoglobulin serum proteins. Less than 1% cross reactivity to bovine, chicken, human, mouse, rabbit and sheep IgG was detected. This antibody may cross react with IgG from other species. Tested for immunofluorescence by the manufacturer. Images for HeLa cells are provided on the manufacturer's website.
Goat anti-rabbit AF647 (ThermoFisher A21244): Manufacturer's statement on antibody testing for IF in human cells: Immunofluorescence analysis of Goat anti-Rabbit IgG (H+L) Cross-Adsorbed Secondary Antibody, Alexa Fluor® 647 conjugate was performed using HeLa cells stained with alpha Tubulin Rabbit Polyclonal Antibody (Product # PA5-16891). The cells were fixed with 4% paraformaldehyde for 10 minutes, permeabilized with 0.1% Triton™ X-100 for 10 minutes, blocked with 1% BSA for 1 hour and labeled with 2 µg/mL primary antibody for 3 hours at room temperature. Goat anti-Rabbit IgG (H+L) Cross-Adsorbed Secondary Antibody, Alexa Fluor® 647 conjugate (Product # A-21244) was used at a concentration of 4 µg/mL in phosphate buffered saline containing 0.2% BSA for 45 minutes at room temperature, for detection of alpha Tubulin in the cytoplasm (Panel a: red). Nuclei (Panel b: blue) were stained with DAPI in SlowFade® Gold Antifade Mountant (Product # S36938). F-actin was stained with Alexa Fluor® 488 Phalloidin (Product # A12379), 1:300) (Panel c: green). Panel d represents the composite image. No nonspecific staining was observed with the secondary antibody alone (panel f), or with an isotype control (panel e). The images were captured at 60X magnification.

Eukaryotic cell lines

Policy information about cell lines and Sex and Gender in Research

Cell line source(s)

HEK293T (CRL-3216), MEFs (CRL-2991) and Drosophila S2 (CRL-1963) were obtained from ATCC.

ex1GFPd2::Cas9 (RC9) mouse embryonic stem cells were used that carry a destabilized GFP-reporter for Rex1-expression and a stably expressed Cas9 transgene integrated into the Rosa26 locus (doi: 10.1038/ncb2267; doi: 10.1016/j.celrep.2018.06.027).
Generation of Phf3 WT and KO mouse embryonic stem cell lines is described in https://doi.org/10.1038/s41467-021-26360-2
Generation of Dido heterozygous KO mouse embryonic stem cell line is described in doi:10.15252/embj.2020105776

HEK293T cell lines generated through CRISPR/Cas9 editing
cell line generation described in https://doi.org/10.1038/s41467-021-26360-2
HEK293T PHF3 KO
HEK293T PHF3 ΔSPOC
HEK293T PHF3 WT
HEK293T PHF3-GFP

Cell line generation described in https://doi.org/10.1038/s41467-023-35853-1
HEK293T DIDO full KO
HEK293T DIDO ΔSPOC

Cell line generation described in this paper:
HEK293T DIDO N[1-88]-Isoform KO
HEK293T DIDO Long Isoform KO
PHF3 ΔSPOC DIDO Full KO
PHF3 KO DIDO Full KO
PHF3 ΔSPOC DIDO ΔSPOC
PHF3 KO DIDO ΔSPOC
DIDO ΔIDR
DIDO 3' AID-GFP
PHF3 ΔSPOC DIDO 3' AID-GFP
PHF3 KO DIDO 3' AID-GFP
DIDO ΔSPOC 3' AID-GFP
DIDO ΔIDR 3' AID-GFP
PHF3 ΔIDR-3xFLAG-mScarlet
PHF3-GFP DIDO full KO
DIDO 3' AID-GFP PHF3-3xFLAG-mScarlet

Cell lines generated through stable overexpression, cell line generation described in this paper:
PHF3 WT / SPOC OE
PHF3 WT / PHF3 OE
PHF3 WT / PHF3 ΔSPOC OE
PHF3 KO / SPOC OE
PHF3 KO / PHF3 OE
PHF3 KO / PHF3 ΔSPOC OE

Authentication	mESCs were authenticated by measuring the expression of known pluripotency marker genes by qPCR. The other cell lines used in this study were not authenticated.
Mycoplasma contamination	All cell lines used in this study were regularly tested for Mycoplasma contamination in a PCR-based assay and tested negative.
Commonly misidentified lines (See ICLAC register)	No commonly misidentified cell lines were used in this study

Plants

Seed stocks	Not applicable, because no plants were used in this study.
Novel plant genotypes	Not applicable, because no plants were used in this study.
Authentication	Not applicable, because no plants were used in this study.

Flow Cytometry

Plots

Confirm that:

☒ The axis labels state the marker and fluorochrome used (e.g. CD4-FITC).

☒ The axis scales are clearly visible. Include numbers along axes only for bottom left plot of group (a 'group' is an analysis of identical markers).

☐ All plots are contour plots with outliers or pseudocolor plots.

☒ A numerical value for number of cells or percentage (with statistics) is provided.

Methodology

Sample preparation	For cell cycle analysis 70-80% confluent cells from 6 cm dish were harvested, the pellet resuspended in 1xPBS and spun down for 5 min with 500xg at 4°C. The pellet was washed again with PBS, resuspended in 800 µL PBS and 2.2 mL cold methanol, gently mixed by inverting the tube and incubated at -20°C o/n. The cells were spun down at 500xg for 5 min, washed with PBS and incubated with 500 µL propidium iodide (PI) buffer (50 µg/mL PI, 10 mM Tris pH 7.5, 5 mM MgCl2, freshly added 200 µg/mL RNAse A) at 37°C for at least 30 min before FACS measurements.<br>For FACS sortings during cell line generation cells were trypsinized, centrifuged at 500g for 5min, resuspended in FACS medium (DMEM + 50% FBS) and passed into FACS tubes through the cell strainer in the tube cap.
Instrument	Measurements for cell cycle analysis were performed on a Bio-Rad ZE5 cell analyzer (12004279, Bio-Rad) with excitation laser at 561 nm (50 mW, PI excitation) and a flow rate of 0.1 µL/sec.<br>FACS sortings during cell line generation were performed in a FACSMelody cell sorter (BD), serial number R6627540033.
Software	The Bio-Rad ZE5 was operated by ZE5 Everest software (version 2.5.0.10, Bio-Rad). Cell cycle data were analyzed in FlowJo (version 10.8.1).<br>The BD FACSMelody cell sorter was operated by BD FACSChorus software (version 1.1.20.0).
Cell population abundance	In FACS-based cell cycle analysis experiments, abundance of cells in G1, S, and G2/M phases of the cell cycle was assessed. In the individual samples, G1 population abundance was 45-65% depending on the genotype, S population abundance was 23-39% depending on the genotype and G2/M abundance was 2-25% depending on the genotype.<br><br>FACS was used for cell sorting during cell line generation for the following purposes:<br>- Sorting of GFP-positive cells 48-72h after transfection with pX458 Cas9-EGFP plasmids to enrich for transfected cells. The abundance of the sorted population was typically between 10% and 80% of the total population depending on transfection efficiency.<br>- Sorting if GFp-negative cells 1-2 weeks after transfection with pX458 Cas9-EGFP plasmids to exclude cells with persistent Cas9 expression. The abundance of the sorted population (GFP-/mScarlet-) was typically >90%.<br>- Sorting of GFP-positive or mScarlet-positive cells during generation of endogenously tagged cell lines. The abundance of the sorted populations (GFP+ low or mScarlet+ low) was typically 0.5% to 10% of the total population depending on the editing efficiency and whether previous enrichment steps (antibiotic selection) had been conducted.<br>Purity of the post-sort fractions was not assessed because single cell sorting was performed and clonal cell populations were validated on the genomic (genotyping PCR, Sanger sequencing) and protein level (Western Blot) before proceeding with experiments.
Gating strategy	For FACS-based cell cycle analysis cells were gated into G1, S and G2/M phase based on their DNA content (propidium iodide signal) using FlowJo (version 10.8.1). Histograms illustrating the gating strategy are shown in Supplementary Figures 23 and

24.

For FACS during cell line generation, singlet population was defined by forward vs. side scatter (FSC vs. SSC) gating. Within the singlet population, populations were gated based of their GFP or mScarlet fluorescence (depending on the editing approach): GFP-/mScarlet- (negative), GFP+ high (exogenous GFP-expression after transfection with pX458 plasmids), GFP+ low (endogenous GFP-expression, generation of endogenously GFP-tagged cell lines) and mScarlet+ low (endogenous mScarlet expression, generation of endogenously mScarlet-tagged cell lines).

☒ Tick this box to confirm that a figure exemplifying the gating strategy is provided in the Supplementary Information.
